# Unraveling the Effects and Characteristics of Proliferating Tumor and Cytotoxic T Cells in Colorectal Cancer

**DOI:** 10.1158/1078-0432.CCR-25-2026

**Published:** 2025-11-07

**Authors:** Meeri Kastinen, Jouni Härkönen, Päivi Sirniö, Hanna Elomaa, Henna Karjalainen, Ville K. Äijälä, Vilja V. Tapiainen, Onni Sirkiä, Vesa-Matti Pohjanen, Maarit Ahtiainen, Olli Helminen, Erkki-Ville Wirta, Jukka Rintala, Sanna Meriläinen, Juha Saarnio, Tero Rautio, Toni T. Seppälä, Jan Böhm, Jukka-Pekka Mecklin, Anne Tuomisto, Markus J. Mäkinen, Juha P. Väyrynen

**Affiliations:** 1 Translational Medicine Research Unit, Medical Research Center Oulu, Oulu University Hospital, University of Oulu, Oulu, Finland.; 2 Department of Pathology, Hospital Nova of Central Finland, Well Being Services County of Central Finland, Jyväskylä, Finland.; 3 Faculty of Health Sciences, A.I. Virtanen Institute for Molecular Sciences, University of Eastern Finland, Kuopio, Finland.; 4 Research Program in Systems Oncology, University of Helsinki, Helsinki, Finland.; 5 Department of Environmental and Biological Sciences, University of Eastern Finland, Kuopio, Finland.; 6 Central Finland Biobank, Hospital Nova of Central Finland, Well Being Services County of Central Finland, Jyväskylä, Finland.; 7 Department of Gastrointestinal Surgery, Oulu University Hospital, Oulu, Finland.; 8 Department of Gastroenterology and Alimentary Tract Surgery, Tampere University Hospital, Tampere, Finland.; 9 Faculty of Medicine and Health Technology, Tampere University and Tays Cancer Centre, Tampere University Hospital, Tampere, Finland.; 10 Department of Gastrointestinal Surgery, Helsinki University Central Hospital, University of Helsinki, Helsinki, Finland.; 11 Applied Tumor Genomics, Research Program Unit, University of Helsinki, Helsinki, Finland.; 12 Faculty of Sport and Health Sciences, University of Jyväskylä, Jyväskylä, Finland.; 13 Department of Education and Research, Wellbeing Services County of Central-Finland, Jyväskylä, Finland.

## Abstract

**Purpose:**

The prognostic role of tumor proliferation in colorectal cancer has been unclear, whereas T-cell proliferation has been associated with favorable outcomes. We investigated characteristics and prognostic significance of proliferating tumor and cytotoxic T cells.

**Experimental Design:**

Two independent colorectal cancer cohorts comprising 1,839 patients were analyzed using multiplex IHC for MKI67 (Ki-67), CD8, and CK. Densities and spatial localization of MKI67^+^ and MKI67^−^ cytotoxic T cells and tumor proliferation rate were assessed via digital image analysis. Single-cell RNA sequencing data from 62 colon cancers were used to characterize proliferating and nonproliferating cells.

**Results:**

High MKI67+ tumor cell percentage was associated with better cancer-specific survival, an antitumorigenic immune microenvironment, downregulation of epithelial–mesenchymal transition, and upregulation of MYC signaling. In the larger cohort, the multivariable HR for high versus low proliferation rate was 0.60 (95% confidence interval, 0.43–0.83). MKI67^+^CD8^+^ T cells exhibited high expression of effector molecules such as GZMB and IFNG and stronger association with favorable prognosis than MKI67^−^CD8^+^ T cells. The multivariable HR for high versus low MKI67^+^CD8^+^ T-cell density was 0.49 (95% confidence interval, 0.35–0.70). However, spatial analysis of tumor cell–T cell co-localization indicated comparable prognostic significance for both subsets when considering their proximity to tumor cells.

**Conclusions:**

Tumor cell proliferation is a marker for better prognosis in colorectal cancer. Although proliferating cytotoxic T cells demonstrate stronger prognostic value than nonproliferating cytotoxic T cells, spatial proximity to tumor cells diminishes this difference. These findings provide new insights into the interplay between tumor proliferation, immune response, and patient outcomes in colorectal cancer.

**Translational Relevance**

Proliferation of tumor and CD8^+^ T cells was analyzed in two extensive colorectal cancer cohorts using multiplex IHC. Signaling pathways associated with proliferation were studied with single-cell RNA sequencing. Tumor cell proliferation proved to be a marker for better prognosis in colorectal cancer and associated with an antitumorigenic immune microenvironment and downregulation of epithelial–mesenchymal transition. Proliferating CD8^+^ T cells had stronger association with better survival than nonproliferating CD8^+^ T cells; however when considering their spatial proximity to tumor cells, the difference in their prognostic ability was diminished. Proliferating CD8^+^ T cells were localized closer to tumor cells and exhibited higher expression of effector molecules. This study provides new insights into the role of tumor cell and T-cell proliferation in the colorectal cancer microenvironment, highlighting potential avenues for refining prognostic biomarkers that consider both tumor and immune cell dynamics.

## Introduction

Colorectal cancer is the third most common cancer worldwide, with an increasing incidence, especially among young adults (1). The tumor microenvironment, a dynamic network shaped by interactions between immune cells and tumor cells, plays a critical role in cancer progression (2). Despite advancements in our understanding of colorectal cancer, current classification systems fail to fully account for the complexity and heterogeneity of the disease. This highlights the need for deeper investigation into the biological features of tumors and their interplay with the surrounding microenvironment.

Tumor cell proliferation rate, often assessed via MKI67 (Ki-67) IHC, is a well-established adverse prognostic feature for many tumor types such as breast cancer (3) and gastrointestinal neuroendocrine tumors (4). However, its prognostic significance in colorectal cancer is controversial (5–8). Notably, most studies have relied on subjective, visual evaluation of conventional single-color IHC slides. Additionally, the relationship between tumor proliferation and patient outcomes may vary depending on the molecular tumor features or treatments received by the patients (9).

In addition to tumor cells, immune cells undergo proliferation within the tumor microenvironment. Among these, cytotoxic CD8^+^ cells are a fundamental part of the antitumor response through their ability to directly eliminate tumor cells (10). High CD8^+^ T-cell density is an established favorable prognostic factor in colorectal cancer (11). However, chronic antigen exposure can lead to T-cell exhaustion, diminishing their cytotoxic potential (12, 13). In contrast, proliferating CD8^+^ cells have been linked to a more active tumor microenvironment in various cancers (14). Nevertheless, larger studies with spatially resolved analysis are needed to comprehensively evaluate the significance of proliferating and nonproliferating cytotoxic T cells in colorectal cancer.

In this study, we aimed to thoroughly evaluate the role of tumor cell proliferation and cytotoxic T-cell proliferation in colorectal cancer using multiplex IHC with digital image analysis. We also analyzed the signaling pathways associated with tumor cell proliferation and the characteristics of proliferating (vs. nonproliferating) cytotoxic T cells using single-cell RNA sequencing (scRNA-seq) data from 62 patients with colon cancer. Based on a prior meta-analysis, we hypothesized that high tumor cell proliferation might be associated with poor survival (15). However, our results link low tumor cell proliferation with worse prognosis, epithelial–mesenchymal transition (EMT), and an immunosuppressive microenvironment. Conversely, proliferating cytotoxic T cells were linked to favorable prognosis and active antitumor cytotoxic mechanisms, supporting our initial hypothesis.

## Materials and Methods

### Patients

Histologic samples of two patient cohorts were analyzed. Cohort 1 consisted of 1,343 patients who underwent surgery at Central Finland Central Hospital in 2000 to 2015 (16). Cohort 2 included 1,011 patients who underwent surgery at Oulu University Hospital in 2006 to 2020 (17, 18). Clinical data were collected from patient records. All patients had surgical resection of the tumor with either curative or palliative indication. The representativeness of the cohorts is analyzed in Supplementary Table S1. Patients who received preoperative radio- or chemotherapy were excluded from the analysis (*N* = 243 in cohort 1 and *N* = 235 in cohort 2). Additionally, patients lacking data on CD8^+^ cells or tumor cell proliferation were excluded (*N* = 12 in cohort 1 and *N* = 24 for cohort 2). Ultimately, 1,088 patients from cohort 1 and 752 patients from cohort 2 were included in the analysis. Supplementary Figure S1 summarizes patient inclusion and exclusion for both cohorts and indicates the analysis-specific *N* used in each table and figure.

Patients who died in less than 30 days after surgery were excluded from survival analysis (*N* = 37 in cohort 1 and *N* = 5 in cohort 2). Follow-up time was limited to 10 years. The median follow-up time among those with no events was 10 years (IQR, 7.3–10.0) in cohort 1 and 7 years (IQR, 4.7–10.0) in cohort 2. The survival analysis included 293 colorectal cancer deaths of 526 total deaths in cohort 1 and 150 colorectal cancer deaths of 273 total deaths in cohort 2. The primary endpoint was cancer-specific survival, defined as the time from surgery to colorectal cancer death or the end of follow-up.

Research was done in accordance with the Declaration of Helsinki. Cohort 1 analyses were approved by the Regional medical research ethics committee of the Wellbeing services county of Central Finland (Dnro 13U/2011, 1/2016, 8/2020, and 2/2023), the Finnish Medicines Agency (Fimea; FIMEA/2023/001573), and the Central Finland Biobank (BB23-0172). Cohort 2 studies were conducted under permission from the Regional medical research ethics committee of the Wellbeing services county of North Ostrobothnia (25/2002, 42/2005, 122/2009, and 37/2020), Fimea (FIMEA/2022/001941), and Biobank Borealis (BB-2017_1012). Participants gave written informed consent for the study in cohort 2. For cohort 1, the need to obtain informed consent from the study patients was waived (Dnro FIMEA/2023/001573).

Digital images of hematoxylin and eosin–stained sections of the tumors had been previously reevaluated for grade (World Health Organization 2019 criteria), tumor budding (International Tumor Budding Consensus Conference criteria; ref. 19), and lymphovascular invasion (16, 17). IHC had been used to determine mismatch repair (MMR) status (MLH1, MSH2, MSH6, and PMS2), *BRAF*^V600E^ status, TP53 expression status, and MYC expression status (16, 20–22). In cohort 1, three multiplex IHC assays combined with supervised machine learning–based analysis had been used to detect CD3^+^ T lymphocytes, CD20^+^CD79A^+^ B lymphocytes, CD20^−^CD79A^+^ plasma cells, M1-like and M2-like macrophages, CD14^+^HLA–DR^+^ mature monocytic cells, CD14^+^HLA–DR^−^ immature monocytic cells, CD66B^+^ granulocytes, and tryptase+ mast cells (23, 24). These assays were based on iterative cycles of staining, scanning, and antibody/chromogen stripping, utilizing 3-amino-9-ethylcarbazole as the chromogen (23, 24). Macrophages were categorized based on a polarization index using four polarization markers (CD86 and HLA–DR for M1 and CD163 and CD206 for M2; ref. 24).

### Multiplex IHC and image analysis

**Figure 1.**
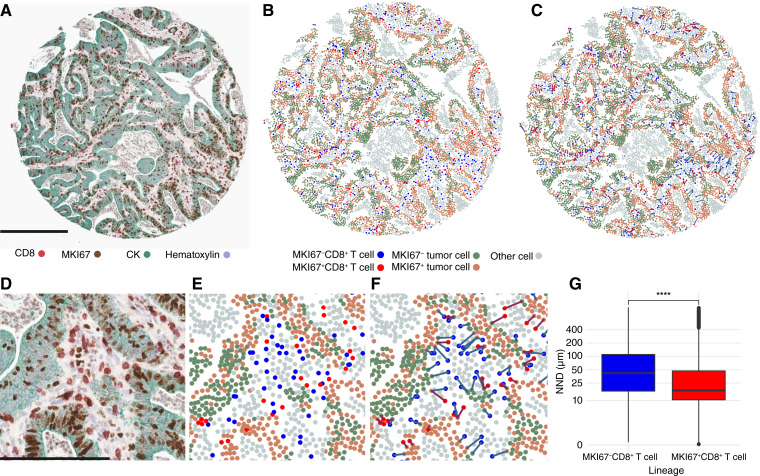
Multiplexed IHC assay for evaluating tumor cell and cytotoxic T-cell proliferation. **A,** An example multiplex IHC image. Scale bar corresponds to 300 μm. **B,** Cell phenotype map. **C,** Nearest neighbor analysis from each CD8^+^ T cell to the nearest tumor cell. **D–F,** Close-ups of the multiplex IHC image, cell phenotype map, and nearest neighbor map. The scale bar corresponds to 150 μm. **G,** Boxplot presenting the distribution of nearest neighbor distances (NND) from each MKI67^−^ (*N* = 1,251,837) and MKI67^+^ (*N* = 337,174) cytotoxic T cell to the closest tumor cell across all tumors. ****, *P* < 0.001.

Tissue microarrays were used in IHC analyses. They were designed to include four cores per tumor (radius 0.5 mm), two from the center of the tumor and two from the invasive margin. A multiplex IHC assay was optimized to evaluate tumor cell and cytotoxic T-cell proliferation. The assay included markers for tumor cells [cytokeratin (CK)], cytotoxic T cells (CD8), and proliferation (MKI67; Fig. 1; Supplementary Table S2). The assay was performed using a cyclic method automated on a Leica BOND RX research stainer (RRID: SCR_025548). In the first cycle, antigen retrieval was conducted using BOND Epitope Retrieval Solution 2 (Leica, AR9640; for 30 minutes at 95°C). CD8 antibodies (Leica, 4B11, RRID: AB_3676740, 1:100) were applied and detected using the BOND Polymer Refine Red Detection Kit (Leica DS9390). In the second cycle, prior antibodies were removed with BOND Epitope Retrieval Solution 1 (Leica, AR9961; for 30 minutes at 95°C). The sections were then incubated with MKI67 antibodies (Cell Marque SP6, RRID: AB_1158037, 1:100) that were detected using the BOND Polymer Refine Detection Kit (Leica DS9800). In the third cycle, previous antibodies were again removed with BOND Epitope Retrieval Solution 1 (Leica, AR9961; for 30 minutes at 95°C), and CK antibodies (Leica, AE1/AE3, RRID: AB_2924990, 1:100) were applied and detected using the BOND Polymer Refine Detection kit, with Green Chromogen (Leica DC9913) replacing DAB as the chromogen. Leica Aperio AT2 (RRID: SCR_021256) was used to scan the slides at 20× objective magnification. In an additional three-plex assay to assess cytotoxic activity, granzyme B (GZMB) was combined with CD8 and MKI67. In this assay, MKI67 antibodies (cycle 1, Cell Marque SP6, 1:100), GZMB antibodies (cycle 2, Cell Signaling Technology, D6E9W, RRID: AB_2799313, 1:30), and CD8 antibodies (cycle 3, Leica, 4B11, 1:100) were applied using the same protocol as the main assay.

QuPath software (version 0.4.3, RRID: SCR_018257) was utilized in image analysis (25). Tissue microarray cores were annotated using the TMA dearrayer tool, and cores with staining artefacts or inadequate tumor epithelium were excluded. A random trees pixel classifier was trained using the Train pixel classifier tool to distinguish between tumor epithelium, stroma, and background. Cell detection settings were first optimized in small annotations of tumors with varying morphologies. Haralick texture features and smoothed features (using 25-μm radius) were calculated for each cell. An object classifier was trained using the random trees method by annotating examples of MKI67^−^ and MKI67^+^ subpopulations of tumor cells (CK^+^) and cytotoxic T cells (CD8^+^), placing remaining cells (CD8^−^CK^−^) in “other” category. For the GZMB−CD8−MKI67 assay, the cell categories included GZMB^+^MKI67^+^CD8^+^ cells, GZMB^+^MKI67^−^CD8^+^ cells, GZMB^−^MKI67^+^CD8^+^ cells, GZMB^−^MKI67^−^CD8^+^ cells, and CD8^−^ “other” cells.

The resulting data were processed with R statistical programming (R core team, version 4.3.0, RRID: SCR_001905) to calculate T-cell densities and the percentage of MKI67^+^ tumor cells for each case. Spatial analyses were performed using the *spatstat* (3.0-3) package. Nearest neighbor distances were calculated to determine the distance from each cytotoxic T cell to the closest tumor cell. Additionally, G-cross function (G_Tumor:T cell_) values at 20-μm radius were computed, assessing the probability of any tumor cell in the sample having at least one T cell of a specified type within a 20-μm radius. According to prior studies, 20-μm distance likely represents a distance that facilitates close interactions between immune and tumor cells (26, 27).

### scRNA-seq analysis

An scRNA-seq dataset comprising 62 patients with colon cancer was downloaded from Gene Expression Omnibus (GSID: GSE178341). The cohort is independent of cohorts 1 and 2 (no participant overlap). Cell subsets were chosen based on population identities characterized in the associated publication (28). Data were processed with scCustomize (v.2.1.2), Seurat (v.1.3.8), and Harmony (v.1.2.0), including cells with detected features within the range of 200 to 2,500, while excluding cells with high mitochondrial read content (>10%). The data were normalized and scaled with the Seurat NormalizeData and ScaleData functions. Clustering was conducted with the Louvain algorithm, and data visualization was performed with Uniform Manifold Approximation and Projection (UMAP). Samples were separately processed based on the cell identity of epithelial cells or CD8^+^ T cells.

#### Epithelial cells

Primary component dimensions were batch-corrected with Harmony using donor ID as a grouping variable. UMAP was performed with the first 10 principal components. Louvain clustering was conducted with a resolution of 0.2. Marker gene detection was conducted, and gene set enrichment analysis (GSEA) was performed for the log fold change–ranked gene list using the fGSEA R package. To characterize epithelial cell–derived cell cycle markers for colorectal cancer, the single-cell data counts were summed into pseudobulk and separated into two groups based on the fractions of proliferating epithelial cells among all epithelial cells, using the median (∼30% of proliferating cells). Differential expression was conducted with limma (3.58.14) as described in the user manual (Linear Models for Microarray and RNA-Seq Data User’s Guide, page 71, April 22, 2023). GSEA was performed for the log fold change–ranked genes with fGSEA.

#### T lymphocytes

Lymphocytes were processed as described previously, with the following modifications: grouping variables donor ID and library preparation protocol were included in Harmony batch correction, and the cell identities were obtained from the previous publication. In addition, a CD8^+^ T-cell functional trajectory and pseudotime analysis was computed with destiny (v.3.18.0) using standard parameters. To assess shifts in gene expression, changes along the trajectory were studied with linear modeling using the glm function from the stats package, analyzing each gene individually. Top hits considered biologically relevant were visualized with the ComplexHeatmap R package (v.2.18.0). To study associations between CD8^+^ T-cell populations across tumors, cell fractions were computed for each case, for which Spearman correlation coefficients were calculated.

### Bulk mRNA analysis

The Cancer Genome Atlas (TCGA) clinical and preprocessed gene expression and mutation data were downloaded from the NIH GDC PanCanAtlas open-access website (https://gdc.cancer.gov/about-data/publications/pancanatlas). RSEM (RNA-Seq by Expectation Maximization) counts used for consensus molecular subtyping (CMS) were downloaded from the Broad Institute data repository (https://gdac.broadinstitute.org/runs/stddata__2016_01_28/).

Tumor mutational burden was computed as the number of somatic entries in the MAF file per 1,000,000 bases, assuming 30,000,000 base pairs in the human exome. CMS was computed with CMScaller (v.2.0.1), and proliferation was assessed by computing the mean log_2_-transformed gene expression from the following genes: *UBE2T*, *MAD2L1*, *CCNB1*, *C8orf59*, *CKS2*, *TUBA1B*, *MRPS23*, and *KPNA2*.

Gene set enrichment for the Hallmark EMT pathway (29) was computed with fGSEA. For associations between proliferation and other features, the Mann–Whitney U test was used for comparisons between continuous and categorical variables, and Spearman correlation was used for continuous versus continuous relationships.

### Statistical analyses

Statistical analyses were performed with IBM SPSS Statistics for Windows (IBM Corp. version 29.0, RRID: SCR_016479) and R statistical programming. *P* value of < 0.05 was considered statistically significant.

Immune cell densities and MKI67^+^ tumor cell percentage were examined as continuous variables to study associations with clinicopathologic characteristics, employing a Mann–Whitney U test or Kruskal–Wallis test as appropriate. Pearson correlation analysis and multiple linear regression were performed to assess the relationship between MKI67^+^ tumor cell percentage and immune cell densities. Assumptions for linear regression were checked using residual plots, variance inflation factor values, and probability–probability plots. Multivariable linear regression models were adjusted for age (continuous), sex (male or female), tumor location (colon or rectum), stage (I–II or III–IV), MMR status (proficient or deficient), and *BRAF* status (wild type or mutant). A Wilcoxon signed-rank test was used to assess statistical significance for paired continuous data.

For survival analyses, immune cell densities and MKI67^+^ tumor cell percentage were categorized into tertiles (T1–T3, from low to high). The Kaplan–Meier function with a log-rank test and Cox regression were utilized for analysis. Time-dependent variables were used to check the proportional hazards assumption in Cox regression models. Cox regression models were adjusted for age (<65, 65–75, and >75), sex (female or male), stage (I–II, III, or IV), lymphovascular invasion (no or yes), grade (low grade or high grade), tumor budding (grade I, II, and III), year of operation (cohort 1: 2000–2005, 2006–2010, and 2011–2015; cohort 2: 2006–2010, 2011–2015, and 2016–2020), tumor location (proximal colon, distal colon, and rectum), *BRAF* status (wild type or mutant), and MMR status (proficient or deficient). Additionally, ROC curves were used to evaluate the performance of proliferating tumor and CD8^+^ cells in predicting cancer-specific survival.

## Results

### High tumor cell proliferation rate is associated with lower stage, MMR deficiency, and *BRAF* mutation

The multiplex IHC assay for assessing tumor cell proliferation and cytotoxic T-cell proliferation was successfully performed for 1,088 patients with colorectal cancer in cohort 1 and 752 patients in cohort 2 (Fig. 1). The median percentage of proliferating MKI67^+^ tumor cells relative to all tumor cells was 41% in cohort 1 and 43% in cohort 2. High tumor cell proliferation was associated with lower stage, absence of lymphovascular invasion, deficient MMR status, *BRAF* mutation, and lower tumor necrosis percentage (Table 1).

**Table 1.** Patient and tumor characteristics in relation to tumor proliferation rate in cohorts 1 and 2.

Characteristic	Cohort 1 Total *N* 1,088	Median (IQR) MKI67^+^ tumor cell percentage	Cohort 2 Total *N* 752	Median (IQR) MKI67^+^ tumor cell percentage
Sex	​	​	​	​
Male	551 (51%)	41% (27%–56%)	398 (53%)	42% (25%–59%)
Female	537 (49%)	42% (26%–57%)	354 (47%)	44% (26%–63%)
*P* value	​	0.961	​	0.224
Age	​	​	​	​
<65	285 (26%)	40% (24%–54%)	227 (30%)	43% (19%–61%)
65–75	379 (35%)	42% (26%–59%)	276 (37%)	43% (27%–61%)
>75	424 (39%)	41% (28%–57%)	249 (34%)	45% (27%–61%)
*P* value	​	0.181	​	0.474
Tumor location	​	​	​	​
Proximal colon	530 (49%)	43% (28%–60%)	314 (42%)	44 (26%–62%)
Distal colon	403 (37%)	38% (24%–53%)	201 (27%)	41% (23%–60%)
Rectum	155 (14%)	43% (28%–57%)	237 (32%)	44% (25%–61%)
*P* value	​	0.007	​	0.627
Stage	​	​	​	​
I	180 (17%)	45% (31%–60%)	173 (23%)	53% (36%–71%)
II	406 (37%)	45% (30%–60%)	249 (33%)	45% (27%–61%)
III	352 (32%)	37% (24%–53%)	247 (33%)	41% (26%–58%)
IV	150 (14%)	33% (22%–49%)	83 (11%)	26% (9.4%–59%)
*P* value	​	<0.0001	​	<0.0001
WHO grade	​	​	​	​
Low grade	895 (82%)	40% (26%–56%)	644 (86%)	43% (25%–60%)
High grade	193 (18%)	44% (29%–60%)	108 (14%)	45% (26%–68%)
*P* value	​	0.017	​	0.288
Lymphovascular invasion	​	​	​	​
No	847 (78%)	43% (28%–59%)	411 (55%)	49% (29%–65%)
Yes	241 (22%)	33% (21%–48%)	341 (45%)	36% (19%–56%)
*P* value	​	<0.0001	​	<0.0001
Tumor necrosis percentage	​	​	​	​
<3%	249 (23%)	45% (31%–61%)	222 (30%)	47% (29%–67%)
3%–39.9%	768 (71%)	39% (25%–55%)	477 (63%)	41% (23%–59%)
≥40%	71 (6.5%)	38% (26%–51%)	53 (7.0%)	45% (22%–66%)
*P* value	​	0.0006	​	0.010
MMR status	​	​	​	​
MMR proficient	924 (85%)	39% (25%–54%)	633 (84%)	41% (23%–59%)
MMR deficient	164 (15%)	52% (37%–67%)	119 (16%)	59% (34%–72%)
*P* value	​	<0.0001	​	<0.0001
*BRAF* status^a^	​	​	​	​
Wild type	907 (83%)	39% (25%–55%)	648 (86%)	41% (23%–59%)
Mutant	179 (16%)	49% (34%–67%)	104 (14%)	56% (34%–72%)
*P* value	​	<0.0001	​	<0.0001
TP53 IHC^b^	​	​	​	​
Mutated pattern	579 (53%)	40% (26%–54%)	432 (57%)	44% (27%–62%)
Wild-type pattern	508 (47%)	43% (27%–59%)	320 (43%)	42% (20%–60%)
*P* value	​	0.069	​	0.309

Abbreviation: WHO, World Health Organization.

a Data missing from two patients in cohort 1.

b Data missing from one patient in cohort 1.

### High tumor cell proliferation rate is linked to activated *MYC* signaling pathways and inversely associated with EMT

**Figure 2.**
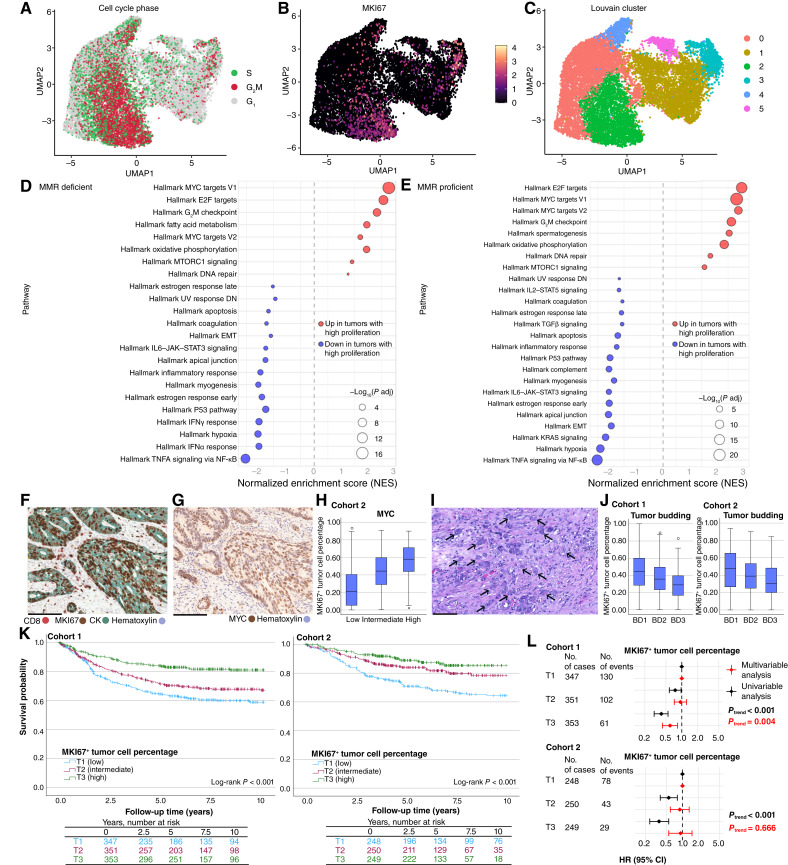
Proliferating tumor cell characteristics. **A–C,** Uniform Manifold Approximation and Projection (UMAP) visualization of tumor cells (*N* = 16,620) based on gene expression in scRNA-seq data from 62 colon cancers. **D** and **E,** GSEA of upregulated and downregulated signaling pathways in MMR-deficient and -proficient tumors with high proliferation rate. **F** and **G,** An example of a tumor with high proliferation rate (MKI67-CD8-CK triple IHC assay) next to serial section of the same tumor demonstrating nuclear MYC staining. **H,** A boxplot showing the distribution of tumor cell proliferation rate in relation to MYC IHC categories in cohort 2 (*N* = 752). **I,** A representative hematoxylin and eosin–stained section showing tumor buds, representing a morphologic feature associated with EMT. **J,** Boxplots showing the distribution of tumor cell proliferation rate according to tumor budding categories (BD1–3, from low to high) in cohorts 1 (*N* = 1,088) and 2 (*N* = 752). **K,** Kaplan–Meier curves for cancer-specific survival for MKI67^+^ tumor cell percentage in cohorts 1 and 2. **L,** Forest plots of uni- and multivariable Cox regression models in cohorts 1 and 2. Multivariable Cox regression models were adjusted for age (<65, 65–75, and >75), sex (female or male), stage (I–II, III, or IV), lymphovascular invasion (no or yes), grade (low grade or high grade), tumor budding (grade I, II, and III), year of operation (cohort 1: 2000–2005, 2006–2010, and 2011–2015; cohort 2: 2006–2010, 2011–2015, and 2016–2020), tumor location (proximal colon, distal colon, or rectum), *BRAF* status (wild type or mutant), and MMR status (proficient or deficient). *P*_trend_ values were calculated by using the three ordinal categories of tumor cell proliferation rate as continuous variables in univariable and multivariable Cox proportional hazards regression models. Scale bars correspond to 100 μm.

scRNA-seq data from 62 patients with colon cancer (28) were analyzed to explore signaling pathways related to tumor cell proliferation. UMAP visualization of tumor cells based on gene expression illustrated that proliferating tumor cells predominantly clustered together in Louvain cluster 2 (Fig. 2A–C).

Considering potential differences in MMR-proficient and -deficient tumors, both groups were separately categorized by their proliferative tumor cell fraction. GSEA demonstrated that both MMR-deficient and -proficient tumors with a high proliferative fraction exhibited upregulation of MYC signaling pathways (Fig. 2D and E). The association between high proliferation and activated MYC signaling was further validated using IHC in cohort 2 (Fig. 2F–H).

EMT pathways were among those downregulated in tumors with high proliferation (Fig. 2E). Aligning with this observation, tumors with high MKI67^+^ percentage showed lower tumor budding, a morphologic feature associated with EMT (Fig. 2I and J; ref. 30).

Differentially expressed genes between high and low proliferative tumors in the scRNA-seq data were then used to generate a proliferative fingerprint. This fingerprint was evaluated in 449 colon cancers from the TCGA dataset, in which it was inversely correlated with EMT pathways (Supplementary Fig. S2), supporting the findings from the scRNA-seq data. The proliferative fingerprint also correlated with tumor mutation burden (*P* = 0.016) and MLH1 methylation (*P* = 0.0071), consistent with the association between tumor cell proliferation and MMR deficiency in cohorts 1 and 2. No significant associations were found with mutations in common colorectal cancer–associated genes such as *APC*, *TP53*, *KRAS*, *NRAS*, *PIK3CA*, *SMAD4*, or *PTEN*.

### High tumor cell proliferation rate is associated with an antitumorigenic immune microenvironment

GSEA of the scRNA-seq data revealed that proliferative tumors displayed downregulation of several proinflammatory signaling pathways such as TNFA signaling via NF-κB and IL6/JAK/STAT3 signaling. To evaluate relationships between tumor cell proliferation and the cellular composition of the tumor microenvironment, three multiplex IHC assays were applied in cohort 1 (Supplementary Fig. S3A–S3F). High MKI67^+^ tumor cell percentage positively correlated with antitumorigenic cell types such as CD3^+^ T cells, M1-like macrophages, and CD14^+^HLA–DR^+^ mature monocytic cells independent of MMR status and other factors (all *P* < 0.001). Conversely, negative correlations were observed with protumorigenic cell types, including M2-like macrophages (*P* = 0.007) and CD14^+^HLA–DR^−^ immature monocytic cells (*P* < 0.001; Supplementary Fig. S3G).

### High tumor cell proliferation rate is associated with better survival

Survival analyses based on MKI67^+^ tumor cell percentage were conducted in cohorts 1 and 2 (Fig. 2K and L; Supplementary Table S3). Kaplan–Meier analysis indicated that patients with a higher tumor cell proliferation rate had better survival compared with those with a lower rate in both cohorts (*P* < 0.001; Fig. 2K). In multivariable Cox regression models, tumor cell proliferation rate was an independent factor for better survival in cohort 1 [HR for T3 vs. T1, 0.60; 95% confidence interval (CI), 0.43–0.83] but did not remain significant in cohort 2 (HR for T3 vs. T1, 0.92; 95% CI, 0.55–1.52; Fig. 2L; Supplementary Table S3). The models were adjusted for multiple clinicopathologic factors such as disease stage, tumor budding, and MMR status. Findings were consistent in sensitivity analysis using a median (above/below) categorization (Supplementary Table S4). The survival associations of tumor cell proliferation rate at the invasive margin and tumor center were largely similar (Supplementary Table S5).

Because prognostic effects may vary by MMR status and stage, we conducted analyses stratified by stage (I–III vs. IV) and MMR status (Supplementary Tables S6 and S7; Supplementary Fig. S4). In the MMR-proficient subset, the findings mirrored those observed in the main analyses, with higher tumor proliferation rate showing association with favorable outcomes in univariable models in both cohorts and in multivariable models in cohort 1 (Supplementary Fig. S4; Supplementary Table S7). In the MMR-deficient subgroup, estimates were directionally similar but underpowered; nonetheless, there was no statistically significant heterogeneity in the association of tumor proliferation rate with survival by MMR status in either cohort (*P*_interaction_ > 0.49). Similarly, there was no evidence that stage modified the prognostic association of tumor cell proliferation rate (*P*_interaction_ > 0.05; Supplementary Fig. S4; Supplementary Table S6).

Additional subgroup analyses evaluating the prognostic significance of tumor cell proliferation according to various tumor and patient characteristics are presented as forest plots in Supplementary Fig. S5. The prognostic significance of tumor cell proliferation was stronger in cases with greater invasion depth (*P*_interaction_ = 0.042) in cohort 1 and in cases with mutated *BRAF* status (*P*_interaction_ = 0.035) in cohort 2. However, no consistent differences were observed across both cohorts.

Adjuvant treatment data were available for cohort 2 patients. Because many systemic treatments preferentially target dividing cells, we tested whether the association between adjuvant treatment status and survival differed by tumor proliferation rate in stage II or III disease. However, there was no statistically significant difference in the benefit associated with adjuvant treatment between high- versus low-/intermediate-proliferation groups in either stage (*P*_interaction_ > 0.36; Supplementary Fig. S6).

### Proliferating cytotoxic T cells exhibit an activated cytotoxic phenotype and localize near tumor cells

Approximately 15% of all CD8^+^ T cells were proliferating (MKI67^+^) in cohort 1 and 18% in cohort 2. Nearest neighbor distance analysis indicated that proliferating CD8^+^ T cells were, on average, located 47% closer to tumor cells than nonproliferating CD8^+^ T cells (Fig. 1G). In both cohorts, proliferating and nonproliferating CD8^+^ T cells were associated with similar clinicopathologic features, including proximal tumor location, lower stage, poor differentiation, absence of lymphovascular invasion, deficient MMR status, and mutant *BRAF* status (Supplementary Table S8). Additionally, in cohort 1, TP53-mutated expression pattern was associated with higher densities of both MKI67^−^CD8^+^ T cells and MKI67^+^CD8^+^ T cells, whereas in cohort 2, older age was associated with higher densities of MKI67^−^CD8^+^ T cells.

**Figure 3.**
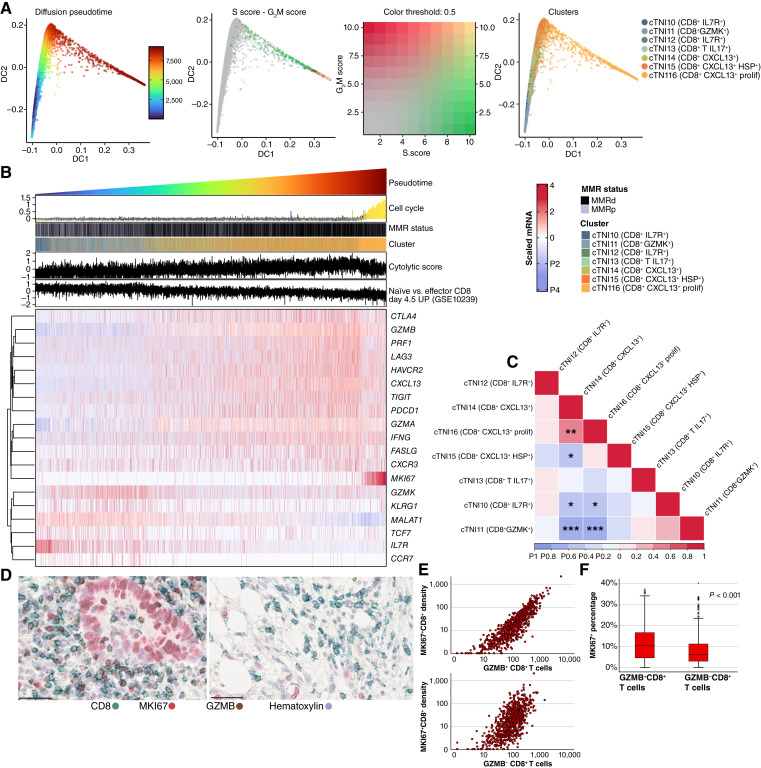
Characteristics of cytotoxic T cells. **A,** Diffusion maps colored by pseudotime, proliferation signaling, and CD8^+^ T-cell clusters. **B,** Diffusion pseudotime in relation to cell cycle score, cytolytic score, and naïve vs. effector CD8 day 4.5 up score, along with a heatmap for genes participating in CD8^+^ T-cell functions in various phases of activation. **C,** Tumor-wise correlation of the relative abundance of various CD8^+^ T-cell clusters. **D,** Representative examples of three-plex IHC (GZMB/CD8/MKI67) images showing cases with high densities of GZMB-positive and -negative cytotoxic cells. **E,** Scatter plot showing the correlation between the densities of MKI67^+^ cytotoxic T cells and GZMB^+^ and GZMB^−^ cytotoxic T cells (*N* = 756 in cohort 2). Scale bar corresponds to 50 μm. **F,** Boxplot of the distribution of MKI67 percentages of CD8^+^ T cells according to GZMB status (*N* = 751 in Cohort 2). *P* value is from a Wilcoxon signed-rank test. **A–C** are based on the scRNA-seq dataset of 62 colon cancer cases. MMRd, MMR deficient; MMRp, MMR proficient. *, *P* < 0.05; **, *P* < 0.01; ***, *P* < 0.001.

To better understand the characteristics of proliferating and nonproliferating CD8^+^ T cells, we utilized pseudotime analysis of cytotoxic T cells in scRNA-seq data from 62 colon cancers (Fig. 3), ordering cells based on their gene expression profiles and enabling inference of their cellular processes along a continuum. In this analysis, proliferating cytotoxic T cells clustered at the end of the pseudotime trajectory, in close proximity to previously characterized CXCL13^+^ activated effector T cells (28), whereas naïve-like cytotoxic T cells were positioned earlier in the pseudotime trajectory (Fig. 3A and B). Proliferating cytotoxic T cells showed high expression of cytolytic genes such as *GZMB* and *PRF1* (31), as well as *IFNG*, a key important antitumor immunoregulatory cytokine. They also exhibited moderate-to-high expression of *FASLG*, which is involved in apoptosis induction, and exhaustion-associated immune checkpoint genes such as *CTLA4*, *PDCD1*, *HAVCR2*, and *LAG3* (28, 32), suggesting a balance between activation and early exhaustion. In contrast, they displayed low expression of *IL7R* and *MALAT1* involved in survival and differentiation of naïve cytotoxic T cells (Fig. 3B). Further highlighting the close association between the proliferation and activation of cytotoxic T cells, the proportion of proliferating cytotoxic T cells was strongly correlated with that of the CXCL13^+^ activated cytotoxic T population (r = 0.52) but showed only weak or negative correlations with CXCL13^–^ cytotoxic T-cell populations (*r* < 0.12; Fig. 3C).

To further investigate the association between proliferating MKI67^+^CD8^+^ T cells and cytotoxic activity, an additional three-plex IHC assay (GZMB−MKI67−CD8) was conducted in cohort 1 (Fig. 3D). This assay demonstrated that a higher proportion of GZMB^+^ cytotoxic T cells were MKI67^+^ compared with GZMB^−^ cytotoxic T cells (*P* < 0.001; Fig. 3F). Moreover, proliferating MK67^+^ cytotoxic T-cell densities showed a stronger correlation with GZMB^+^ cytotoxic T-cell densities (*r* = 0.93) than GZMB^−^ cytotoxic T-cell densities (*r* = 0.42; Fig. 3E). Collectively, these findings suggest that proliferating cytotoxic T cells exhibit an activated cytotoxic phenotype and are closely associated with other activated cytotoxic T cells.

### The prognostic significance of nonproliferating cytotoxic T cells is influenced by their spatial distribution

**Figure 4.**
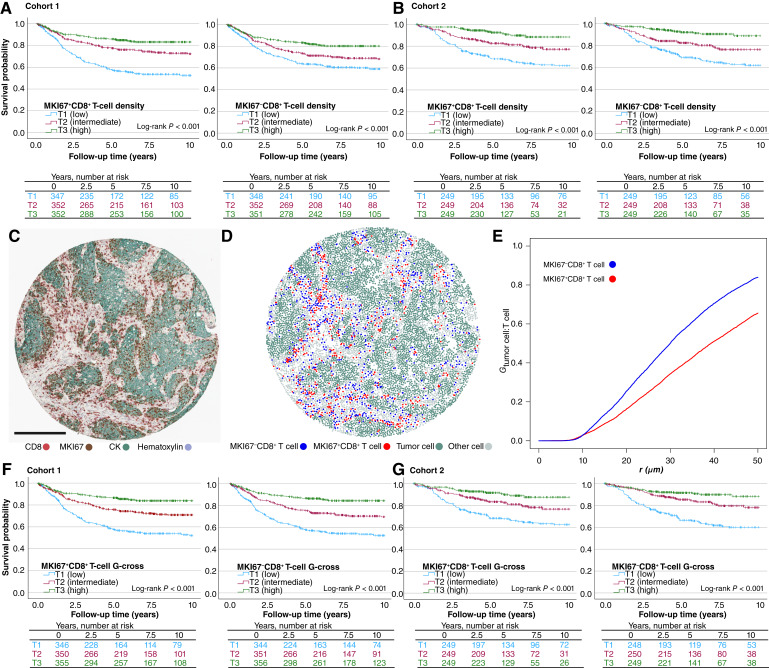
Density and spatial analysis of proliferating and nonproliferating cytotoxic T cells and survival. **A** and **B,** Kaplan–Meier curves for proliferating and nonproliferating cytotoxic T-cell densities in cohorts 1 and 2. **C–E,** G-cross spatial analysis in an example tissue microarray core. **C,** The multiplex IHC image and (**D**) identified CD8^+^ T-cell subsets. Scale bar corresponds to 250 μm. **E,** G-cross functions evaluating the likelihood of tumor cells being co-located with MKI67^−^ and MKI67^+^ cytotoxic T cells within specified radii. **F** and **G,** Kaplan–Meier curves for proliferating and nonproliferating cytotoxic T-cell G-cross function values.

To investigate whether proliferating cytotoxic CD8^+^ cells are more strongly associated with favorable outcomes compared with their nonproliferating counterparts, we conducted Kaplan–Meier analysis and Cox regression models (Fig. 4A and B; Supplementary Tables S9 and S10). In these analyses, high MKI67^+^CD8^+^ T-cell densities were slightly more predictive of better survival than MKI67^−^CD8^+^ T cells. The multivariable HR for high versus low MKI67^+^CD8^+^ T-cell densities was 0.49 (95% CI, 0.35–0.70) in cohort 1 and 0.38 (95% CI, 0.21–0.68) in cohort 2 (Table 2). When compared with overall CD8^+^ T-cell densities, proliferating CD8^+^ T-cell densities also remained a stronger prognostic indicator (Supplementary Table S11). Using a median (above/below) categorization, findings were consistent in cohort 1, but this sensitivity analysis did not confirm a stronger prognostic significance of MKI67^+^CD8^+^ T cells in cohort 2 (Supplementary Table S4).

**Table 2.** Cox regression models for cancer-specific survival according to CD8^+^ T cell densities in cohorts 1 and 2.

​	Cohort 1				Cohort 2			
	No. of cases	No. of events	Univariable HR (95% CI)	Multivariable HR (95% CI)	No. of cases	No. of events	Univariable HR (95% CI)	Multivariable HR (95% CI)
MKI67^+^CD8^+^ T cells	​	​	​	​	​	​	​	​
T1	347	153	1 (referent)	1 (referent)	249	85	1 (referent)	1 (referent)
T2	352	86	0.50 (0.39–0.66)	0.72 (0.55–0.95)	249	45	0.52 (0.36–0.75)	0.75 (0.50–1.13)
T3	352	54	0.30 (0.22–0.41)	0.49 (0.35–0.70)	249	20	0.23 (0.14–0.37)	0.38 (0.21–0.68)
*P*_trend_	​	​	<0.001	<0.001	​	​	<0.001	0.001
MKI67^−^CD8^+^ T cells	​	​	​	​	​	​	​	​
T1	348	130	1 (referent)	1 (referent)	249	81	1 (referent)	1 (referent)
T2	352	100	0.72 (0.55–0.93)	0.74 (0.57–0.97)	249	48	0.58 (0.41–0.83)	0.89 (0.60–1.32)
T3	351	63	0.44 (0.32–0.59)	0.63 (0.46–0.86)	249	21	0.24 (0.15–0.39)	0.44 (0.26–0.76)
*P*_trend_	​	​	<0.001	0.002	​	​	<0.001	0.005

Multivariable Cox regression models were adjusted for age (<65, 65–75, and >75), sex (female or male), stage (I–II, III, or IV), lymphovascular invasion (no or yes), grade (low grade or high grade), tumor budding (grade I, II, and III), year of operation (cohort 1: 2000–2005, 2006–2010, and 2011–2015; cohort 2: 2006–2010, 2011–2015, and 2016–2020), tumor location (proximal colon, distal colon, or rectum), *BRAF* status (wild type or mutant), and MMR status (proficient or deficient).

*P*_trend_ values were calculated by using the three ordinal categories of immune cell densities as continuous variables in univariable and multivariable Cox proportional hazards regression models.

Considering the influence of MMR status on the tumor microenvironment, we performed subgroup analyses stratified by MMR status (Supplementary Table S7; Supplementary Fig. S7). In univariable models, the prognostic significance of MKI67^+^CD8^+^ T cells was stronger in MMR-proficient tumors (univariable *P*_interaction_ = 0.054 for cohort 1 and 0.037 for cohort 2). However, this interaction did not persist after multivariable adjustment in cohort 1 (*P*_interaction_ = 0.399), and reliable multivariable modeling in cohort 2 was not feasible for the MMR-deficient subgroup because of the low number of events. For MKI67^−^CD8^+^ T cells, we found no evidence that prognostic value differed by MMR status (*P*_interaction_>0.36).

We also stratified analyses by stage (I–III vs. IV; Supplementary Table S6; Supplementary Fig. S7). In cohort 1, higher densities of both MKI67^+^CD8^+^ T cells and MKI67^−^CD8^+^ T cells were associated with better survival in stages I to III but not in stage IV (all *P*_interaction_ < 0.05). Cohort 2 showed similar tendencies, but statistically significant interaction was only observed for MKI67^−^CD8^+^ T cells in univariable analysis (*P*_interaction_ = 0.050).

Given the distinct spatial distribution of MKI67^−^CD8^+^ T cells and MKI67^+^CD8^+^ T cells, we further assessed their prognostic value by incorporating spatial localization in the analysis. Using G-cross function, we quantified the likelihood of tumor cells being co-localized (within a 20-μm radius) with these T-cell populations (Fig. 4C–E). Higher G-cross function values for both MKI67^−^CD8^+^ T cells and MKI67^+^CD8^+^ T cells were associated with favorable prognosis (Fig. 4F and G). To benchmark against tumor proliferation rate, we first evaluated ROC performance for MKI67^+^ tumor cell percentage (Supplementary Fig. S8A) and then compared CD8^+^ T-cell densities and G-cross function values for discriminating cancer-specific survival (Supplementary Fig. S8B). The T-cell metrics generally showed slightly higher discriminatory capacity than tumor proliferation rate. For MKI67^−^CD8^+^ T cells, the AUC for G-cross function values was slightly higher than that for MKI67^−^CD8^+^ T-cell densities, whereas for MKI67^+^CD8^+^ T cells, G-cross function values and cell densities demonstrated comparable performance. In Cox regression models, MKI67^+^CD8^+^ T-cell densities remained a stronger prognostic factor than their G-cross function values, whereas for MKI67^−^CD8^+^ T cells, G-cross function values showed stronger prognostic value than cell densities (Supplementary Table S12). Taken together, these findings suggest that proliferating cytotoxic T cells are strongly associated with favorable outcomes irrespective of their spatial localization within the tumor microenvironment. In contrast, for the nonproliferating cytotoxic T cells, their proximity to tumor cells seems to contribute to the prognostic significance.

## Discussion

This study assessed the significance of tumor cell and cytotoxic T-cell proliferation in colorectal cancer by leveraging two large colorectal cancer cohorts with more than 2,000 patients and scRNA-seq data from 62 patients. High tumor cell proliferation was associated with better survival, MMR deficiency, downregulation of EMT, and upregulation of MYC signaling, alongside an antitumorigenic tumor microenvironment. Similarly, proliferating cytotoxic T cells were associated with favorable prognosis, as well as localization near tumor cells and high expression of key cytotoxic molecules such as GZMB. By integrating multimodal data, this study provides insights into the prognostic relevance of tumor and T-cell proliferation in colorectal cancer and highlights potential opportunities for therapeutic targeting and patient stratification. The findings challenge traditional perceptions of tumor proliferation as an adverse feature and emphasize the intricate interplay between tumor growth dynamics and the immune landscape.

MKI67 is a cellular marker of proliferation, appearing in cell cycle phases G_1_, S, G_2_, and M (33, 34). High tumor proliferation rate has been associated with aggressive tumor behavior in many malignancies, and MKI67 IHC is used in the clinical setting in breast cancer prognostic classification (35) and gastroenteropancreatic neuroendocrine tumor classification (36), among others. In colorectal cancer, the prognostic role of tumor proliferation rate has been controversial, with reports showing associations with both worse (5, 8) and better survival (6). Explanations for contradictory result have been discussed, and for instance, deficient MMR status (9) or higher apoptosis rates in proliferating tumors (37) have been suggested to account for the reported associations with better survival. In our study, high tumor cell proliferation was associated with favorable outcomes in both cohorts and showed prognostic value independent of MMR status and other covariates in cohort 1 but did not remain an independent prognostic factor in cohort 2. We considered whether enhanced sensitivity to adjuvant therapy might underlie the favorable prognostic association of high proliferation rate. In cohort 2, however, we found no evidence that patients with high proliferation derived greater benefit from adjuvant treatment, making differential postoperative therapy an unlikely explanation. However, interpretation is limited by nonrandom treatment assignment (selection likely reflected age, comorbidity burden, stage at diagnosis, and tumor characteristics) and by the absence of adjuvant treatment data in cohort 1. Still, the results suggest a need to reconsider the conventional view of tumor proliferation as a negative prognostic indicator across tumor types. Notably, also a recent pathway-level colorectal cancer subtyping study highlighted a slowly proliferating biological phenotype associated with poor clinical outcomes (38), aligning with our observation.

Our study highlights the inverse relationship between tumor proliferation and EMT signaling as a potential explanation for the observed association between higher proliferation rate and better survival. The go-or-grow hypothesis suggests that cancer cells show either a migratory or proliferative phenotype but not both simultaneously (39, 40). According to this concept, tumors with a lower proliferation rate are more invasive and more likely to metastasize (41). This hypothesis has been studied in gliomas and melanoma, among others (40, 42). In colorectal cancer, previous findings have indirectly supported the concept, showing that nonproliferating p16^+^ cells at the invasive margin are associated with invasive tumor growth and poor prognosis (7). Our results provide further support, demonstrating that high tumor cell proliferation correlates with downregulation of EMT signaling, lower tumor budding scores, reduced lymphovascular invasion, and better survival. However, the prognostic significance of proliferation rate at the invasive margin versus tumor center did not considerably differ in our cohorts. The MYC signaling pathway plays a central role in regulating cell proliferation and metabolism (43). In line with this, our GSEA revealed upregulation of MYC signaling in tumors with high proliferation, a finding that was further validated using MYC IHC. Previous colorectal cancer studies conform with our findings (44, 45), and it has been demonstrated that MYC regulates tumor proliferation through various mechanisms, including CLK3 and the Wnt/β-catenin pathway (45, 46).

We found that higher tumor cell proliferation correlated with an antitumorigenic microenvironment, characterized by M1-like macrophage polarization, monocytic cell maturity, and higher T-cell densities. These findings were also independent of MMR status and other confounding factors. Previous studies have linked M2-like macrophages to an immunosuppressive tumor microenvironment and EMT, (47, 48) whereas M1-like macrophages are typically associated with tumor suppression (49). For instance, Kawamura and colleagues (50) recently showed higher M2-like macrophage densities in areas of tumor budding. A key strength of our study was the multimarker approach to assess macrophage polarization (CD86 and HLADR for M1; CD163 and CD206 for M2), providing more accurate characterization compared with single-marker analysis.

Besides tumor cell proliferation, we evaluated the proliferation of cytotoxic T cells and found that proliferating cytotoxic T cells demonstrated stronger prognostic value than nonproliferating cytotoxic T cells. These findings align with previous studies connecting proliferation of cytotoxic cells with favorable tumor characteristics and improved survival (10, 14). Using scRNA-seq data, we further characterized proliferating cytotoxic T cells and identified an activated cytotoxic phenotype, marked by high expression of key effector molecules such as IFNG and GZMB. In contrast, a substantial proportion of nonproliferating cytotoxic T cells exhibited a less activated or exhausted phenotype. A recent study by Küçükköse and colleagues (51) also highlighted the importance of proliferating cytotoxic T cells in MMR-deficient colorectal cancers, showing that nonmetastatic tumors had higher levels of these cells than metastatic tumors.

Proliferating cytotoxic T cells were located closer to tumor cells than nonproliferating cytotoxic T cells. When the spatial localization was accounted for using the G-cross spatial measurement—assessing the likelihood of tumor cells being co-located with cytotoxic T cells within a 20-μm radius—the prognostic distinction between proliferating and nonproliferating cytotoxic T cells diminished. This suggests that the proximity of T cells to tumor cells may play a crucial role in their effector function, potentially compensating for the lack of proliferation in cells located near tumor cells (52, 53). Considering immune cell spatial localization is essential for the development of more precise tumor–immune biomarkers. For example, our previous study demonstrated that the T-cell proximity score, which measures tumor cell–T cell co-localization, provided a stronger prognostic value compared with overall T-cell densities (16).

One of the key limitations of the study is the use of tissue microarrays to evaluate the proliferation status of tumor cells and CD8^+^ T cells as the cores might not be representative of the entire tumor. However, previous studies have successfully examined CD8^+^ T-cell densities and tumor cell proliferation using tissue microarrays (10, 11). Another limitation is the predominantly White Caucasian patient population, which may limit the generalizability of the results and necessitate further validation in other ethnic groups. Data on recurrence-free survival were not available. However, with the long follow-up, cancer-specific survival serves as a practical endpoint for prognosis. Lastly, *KRAS/NRAS* mutation status was unavailable for cohorts 1 and 2. However, *RAS* mutations were not associated with the proliferative fingerprint in the TCGA cohort. Although *RAS* alterations are clinically relevant as predictive biomarkers, their prognostic value seems to be weaker than that of *BRAF* and MMR status, which were included in our adjusted analysis (54). The strengths include two large, well-annotated cohorts from tertiary and secondary treatment centers providing robust clinicopathologic data. The use of multiplex IHC allowed for a detailed, quantitative analysis of tumor cell proliferation, proliferating T-cell densities, and their spatial distribution within the tumor microenvironment. Furthermore, the integration of scRNA-seq data and TCGA data provided comprehensive insights into the molecular features associated with tumor cell and cytotoxic T-cell proliferation in colorectal cancer. Notably, this study is also among the largest so far to incorporate spatial point pattern analysis to characterize immune cell infiltration in colorectal cancer, offering a comprehensive perspective on the role of tumor and cytotoxic T-cell proliferation and co-localization in disease progression.

In conclusion, high tumor cell proliferation is linked to better prognosis, potentially through reduced EMT and an antitumorigenic microenvironment. Additionally, proliferating cytotoxic T cells exhibit an activated phenotype and strong prognostic value, whereas the spatial proximity to tumor cells plays a more critical role in the prognostic significance of nonproliferating cytotoxic T cells. This study enhances our understanding of the role of proliferation in colorectal cancer and highlights potential avenues for refining prognostic biomarkers that consider both tumor and immune cell dynamics.

## Supplementary Material

Supplementary Figure S1 Flow chart representing patient flow in Cohorts 1 and 2.

Supplementary Figure S2 Tumor cell proliferation fingerprint and associations to molecular features in The Cancer Genome Atlas database.

Supplementary Figure S3 The associations of tumor cell proliferation with the composition of tumor microenvironment.

Supplementary Figure S4 Kaplan-Meier curves for MKI67+ tumor cell percentage categorized by stage (stage I-III, IV) and MMR status (proficient, deficient).

Supplementary Figure S5 Subgroup analyses of cancer-specific survival according to MKI67+ tumor cell percentage in Cohort 1 and Cohort 2.

Supplementary Figure S6 Kaplan-Meier curves for adjuvant treatment status in relation to MKI67+ tumor cell percentage in Cohort 2

Supplementary Figure S7 Kaplan-Meier curves for proliferating and non-proliferating CD8+ T cell densities categorized by stage (stage I-III, IV) and MMR status (proficient, deficient) in Cohorts 1 and 2.

Supplementary Figure S8 Receiver-operating characteristics (ROC) analysis for cancer-specific survival.

Supplementary Table S1 Representativeness of the study participants.

Supplementary Table S2 Reagents and resources used in the study methods.

Supplementary Table S3 Multivariable Cox regression analysis for cancer-specific survival according to tumor proliferation rate and covariates in Cohorts 1 and 2.

Supplementary Table S4 Sensitivity analysis of Cox regression models for cancer-specific survival according to tumor cell proliferation and proliferating and non-proliferating CD8+ T cells, using alternative cut-points.

Supplementary Table S5 Cox regression models for cancer-specific survival according to tumor proliferation rate in the invasive margin (im) and tumor center (ct)

Supplementary Table S6 Cox regression models for cancer-specific survival according to tumor cell proliferation and proliferating and non-proliferating CD8+ T cells, stratified by stage (I-III vs. IV)

Supplementary Table S7 Cox regression models for cancer-specific survival according to tumor cell proliferation and proliferating and non-proliferating CD8+ T cells, stratified by mismatch repair status.

Supplementary Table S8 Tumor and patient characteristics in relation to proliferating and non-proliferating cytotoxic T cell densities in Cohorts 1 and 2.

Supplementary Table S9 Multivariable Cox regression analysis for cancer-specific survival according to non-proliferating cytotoxic T cell densities and covariates in Cohorts 1 and 2.

Supplementary Table S10 Multivariable Cox regression analysis for cancer-specific survival according to proliferating cytotoxic T cell densities and covariates in Cohorts 1 and 2.

Supplementary Table S11 Cox regression models comparing the prognostic value of CD8+ T cell densities and overall CD8+ T cell densities in Cohorts 1 and 2.

Supplementary Table S12 Cox regression models comparing the prognostic value of CD8+ T cell densities and G-cross function values in Cohorts 1 and 2.

## Data Availability

An scRNA-seq dataset comprising 62 patients with colon cancer was downloaded from Gene Expression Omnibus (GSID: GSE178341). TCGA clinical and preprocessed gene expression and mutation data were downloaded from the NIH GDC PanCanAtlas open-access website (https://gdc.cancer.gov/about-data/publications/pancanatlas). Other datasets generated and analyzed during this study are not publicly available because of confidentiality of patient data. The sharing of data will require approval from relevant ethics committees and/or biobanks. Further information, including the procedures to obtain and access data of Finnish Biobanks, is described at https://finbb.fi/en/fingenious-service. Requests for data not in the Finnish Biobanks can be directed to the corresponding author. In addition, underlying raw materials (e.g., source data behind graphs, full IHC images, and related raw files) are available from the corresponding author upon reasonable request, subject to the approvals described above.
