# Supplementary figures and images for: Unraveling the Effects and Characteristics of Proliferating Tumor and Cytotoxic T Cells in Colorectal Cancer
Source: Clin Cancer Res. 2025 Nov 7;32(2):350–62. doi: 10.1158/1078-0432.CCR-25-2026 (PMC12809117; doi:10.1158/1078-0432.CCR-25-2026)

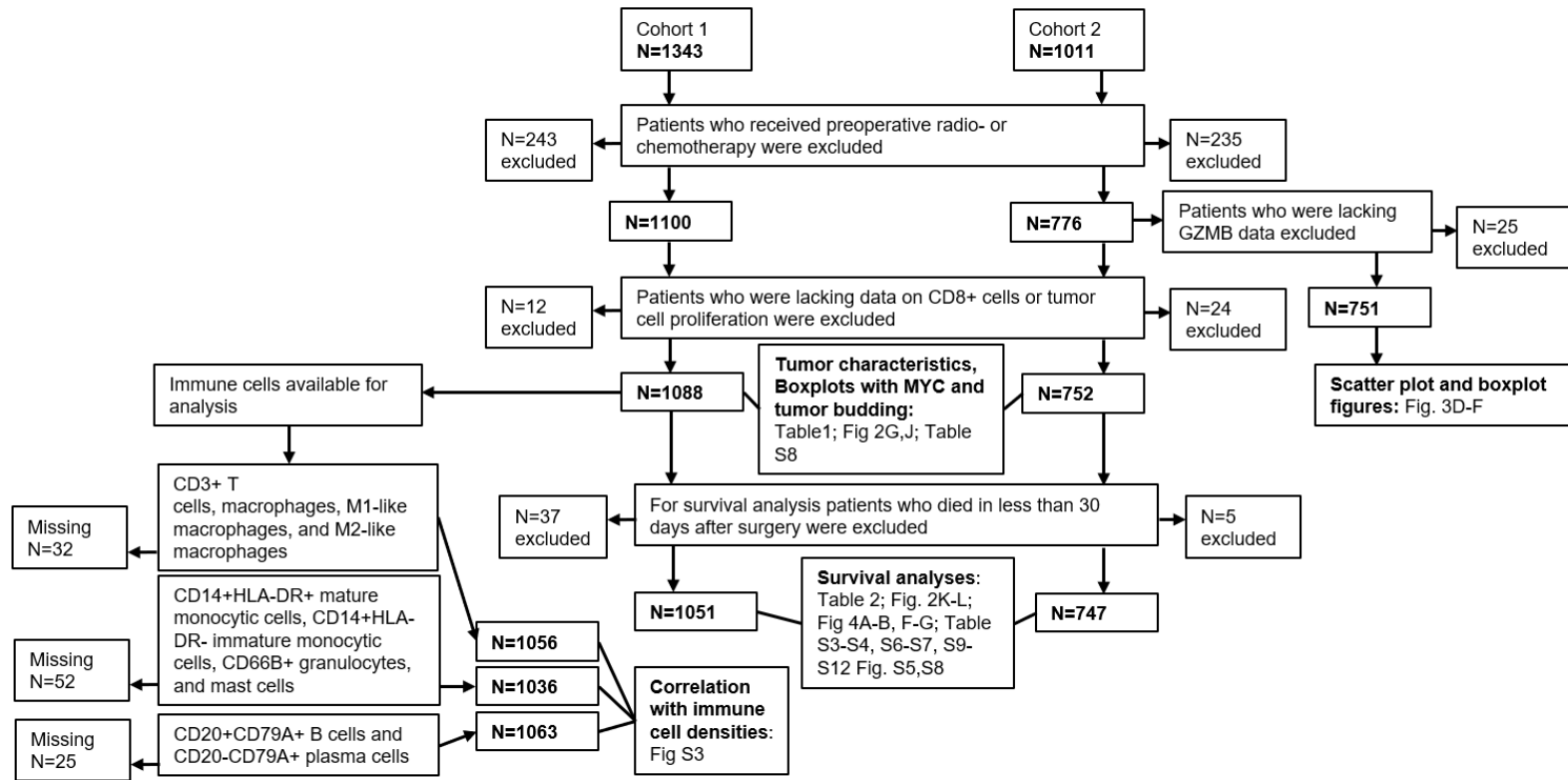

**Figure S1.** Flow chart representing patient flow in Cohorts 1 and 2.

Supplement: Supplementary Figure S1 — Flow chart representing patient flow in Cohorts 1 and 2. [file ccr-25-2026_supplementary_figure_s1_suppfs1.pdf]
