# Supplementary material for: Unraveling the Effects and Characteristics of Proliferating Tumor and Cytotoxic T Cells in Colorectal Cancer
Source: Clin Cancer Res. 2025 Nov 7;32(2):350–62. doi: 10.1158/1078-0432.CCR-25-2026 (PMC12809117; doi:10.1158/1078-0432.CCR-25-2026)
Supplement: Supplementary Figure S2 — Tumor cell proliferation fingerprint and associations to molecular features in The Cancer Genome Atlas database. [file ccr-25-2026_supplementary_figure_s2_suppfs2.pdf]

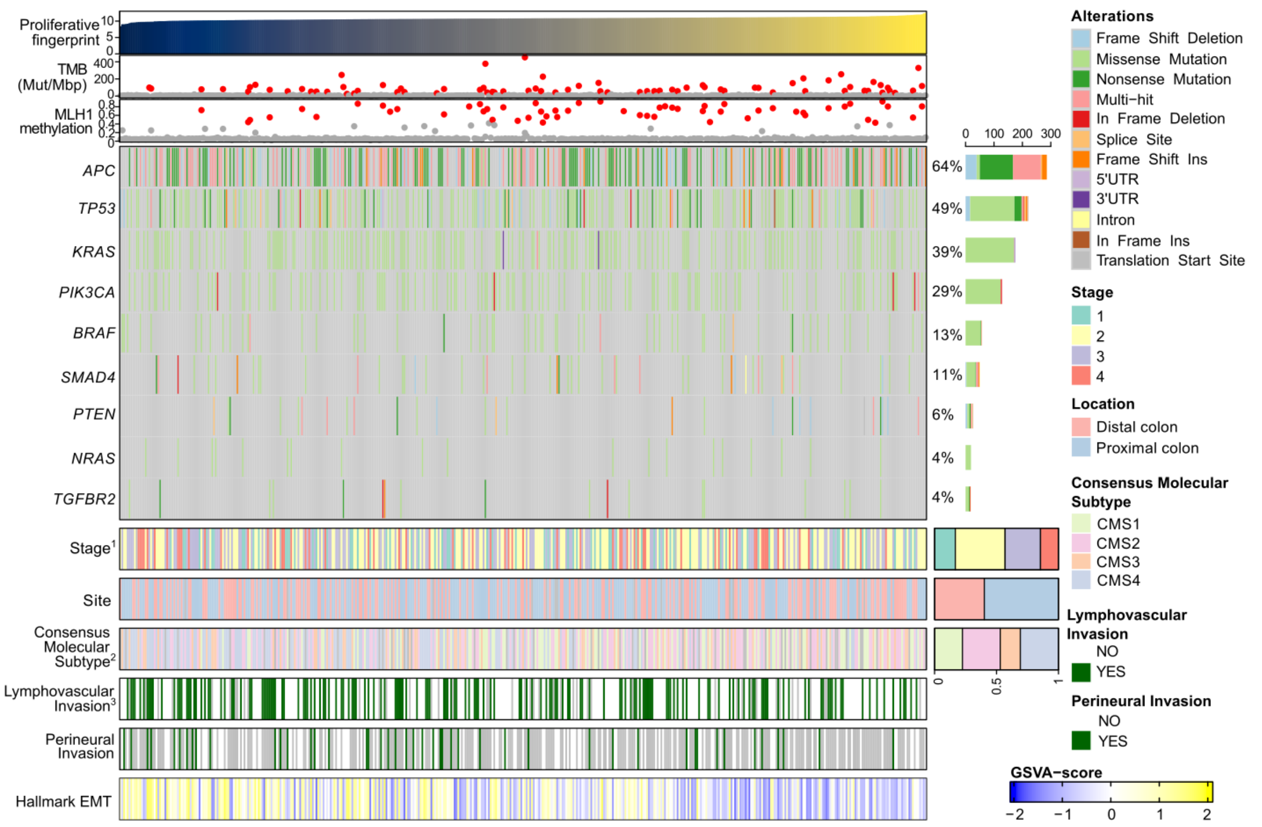

Figure S2. Tumor cell proliferation fingerprint and associations to molecular features in The Cancer Genome Atlas database. Superscripts indicate p values: [1] p=0.008 (local vs. advanced), [2] <0.0005(CMS2 vs. other types) and <0.0001 (CMS1 vs. other types), and [3] p=0.025.
