# Supplementary material for: Unraveling the Effects and Characteristics of Proliferating Tumor and Cytotoxic T Cells in Colorectal Cancer
Source: Clin Cancer Res. 2025 Nov 7;32(2):350–62. doi: 10.1158/1078-0432.CCR-25-2026 (PMC12809117; doi:10.1158/1078-0432.CCR-25-2026)
Supplement: Supplementary Figure S3 — The associations of tumor cell proliferation with the composition of tumor microenvironment. [file ccr-25-2026_supplementary_figure_s3_suppfs3.pdf]

Kastinen et al. Unraveling the effects and characteristics of proliferating tumor and cytotoxic T cells in colorectal cancer

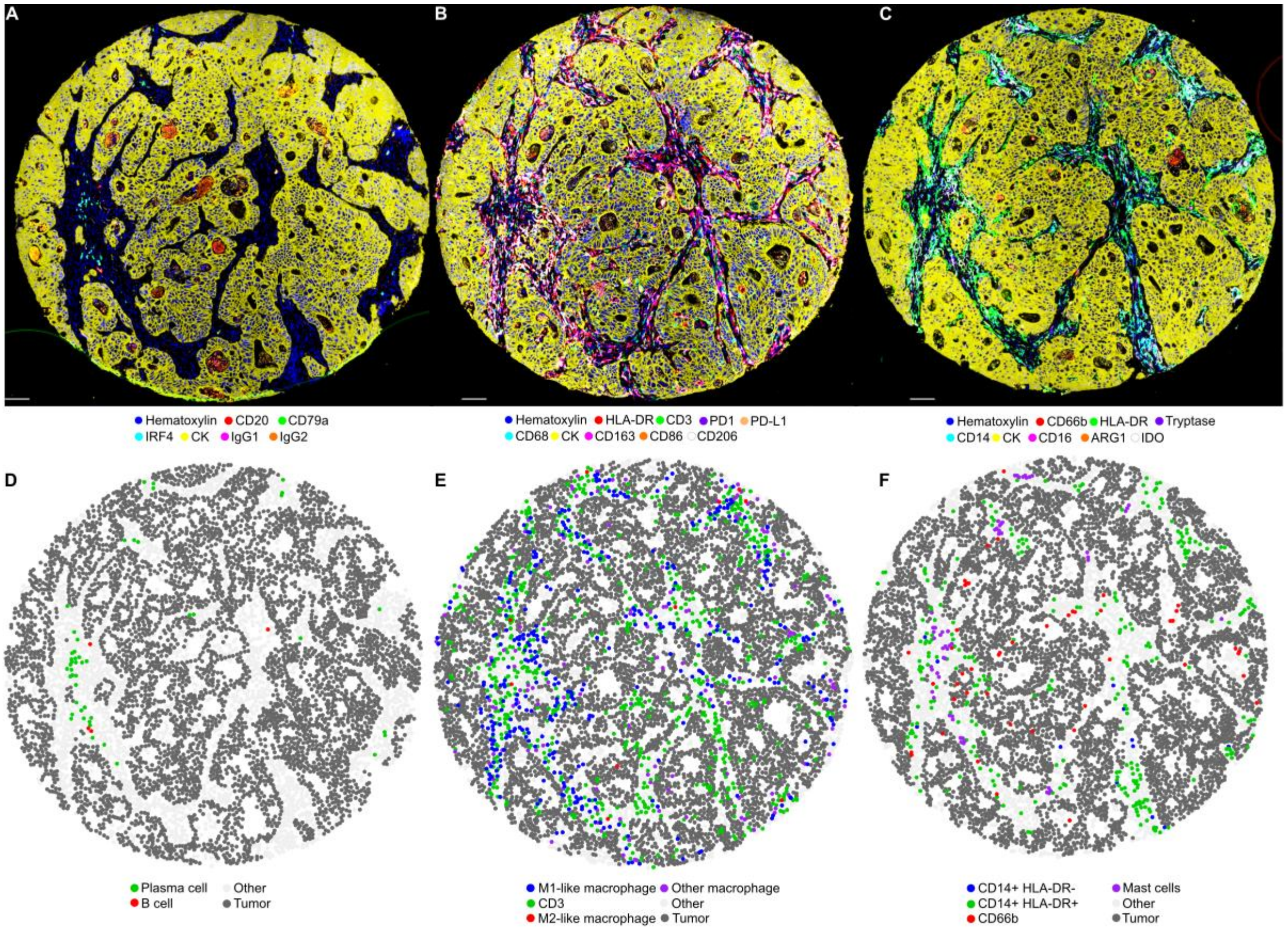

G

	Unadjusted Pearson r	Adjusted Beta
CD79A+CD20+ B cells	0.093**	0.079**
CD3+ T cells	0.270***	0.236***
CD79A+CD20- Plasma cells	0.103***	0.067*
CD68/CD163+ macrophages	0.095**	0.062*
M1-like macrophages	0.218***	0.193***
M2-like macrophages	-0.065*	-0.081**
CD14+HLA-DR+ monocytic cells	0.182***	0.131***
CD14+HLA-DR- monocytic cells	-0.116***	-0.121***
CEACAM8+ (CD66b+) granulocytes	0.102***	0.057
Mast cells	0.060	0.071*

Figure S3. The associations of tumor cell proliferation with the composition of tumor microenvironment. **A-F**. Representative examples of three multiplex immunohistochemistry assays (**A-C**) and image analysis result images (**D-F**). Scale bars correspond to 100 µm. **G.** Correlation between immune cell densities and MKI67+ tumor cell percentage. The adjusted correlation coefficients (Beta) were based on multivariable linear regression models which were adjusted for age (continuous), sex (male, female), localization (colon, rectum), stage (I-II, III-IV), MMR status (proficient, deficient), and *BRAF* status (wild-type, mutant). Asterisks indicate p values: *<0.05, **<0.01, ***<0.001. The analysis was based on Cohort 1; N=1056 for CD3+ T cells, macrophages, M1-like macrophages, and M2-like macrophages; N=1036 for CD14+HLA-DR+ mature monocytic cells, CD14+HLA-DR- immature monocytic cells, CD66B+ granulocytes, and mast cells; N=1063 for CD20+CD79A+ B cells and CD20-CD79A+ plasma cells.
