# Supplementary material for: Unraveling the Effects and Characteristics of Proliferating Tumor and Cytotoxic T Cells in Colorectal Cancer
Source: Clin Cancer Res. 2025 Nov 7;32(2):350–62. doi: 10.1158/1078-0432.CCR-25-2026 (PMC12809117; doi:10.1158/1078-0432.CCR-25-2026)
Supplement: Supplementary Figure S4 — Kaplan-Meier curves for MKI67+ tumor cell percentage categorized by stage (stage I-III, IV) and MMR status (proficient, deficient). [file ccr-25-2026_supplementary_figure_s4_suppfs4.pdf]

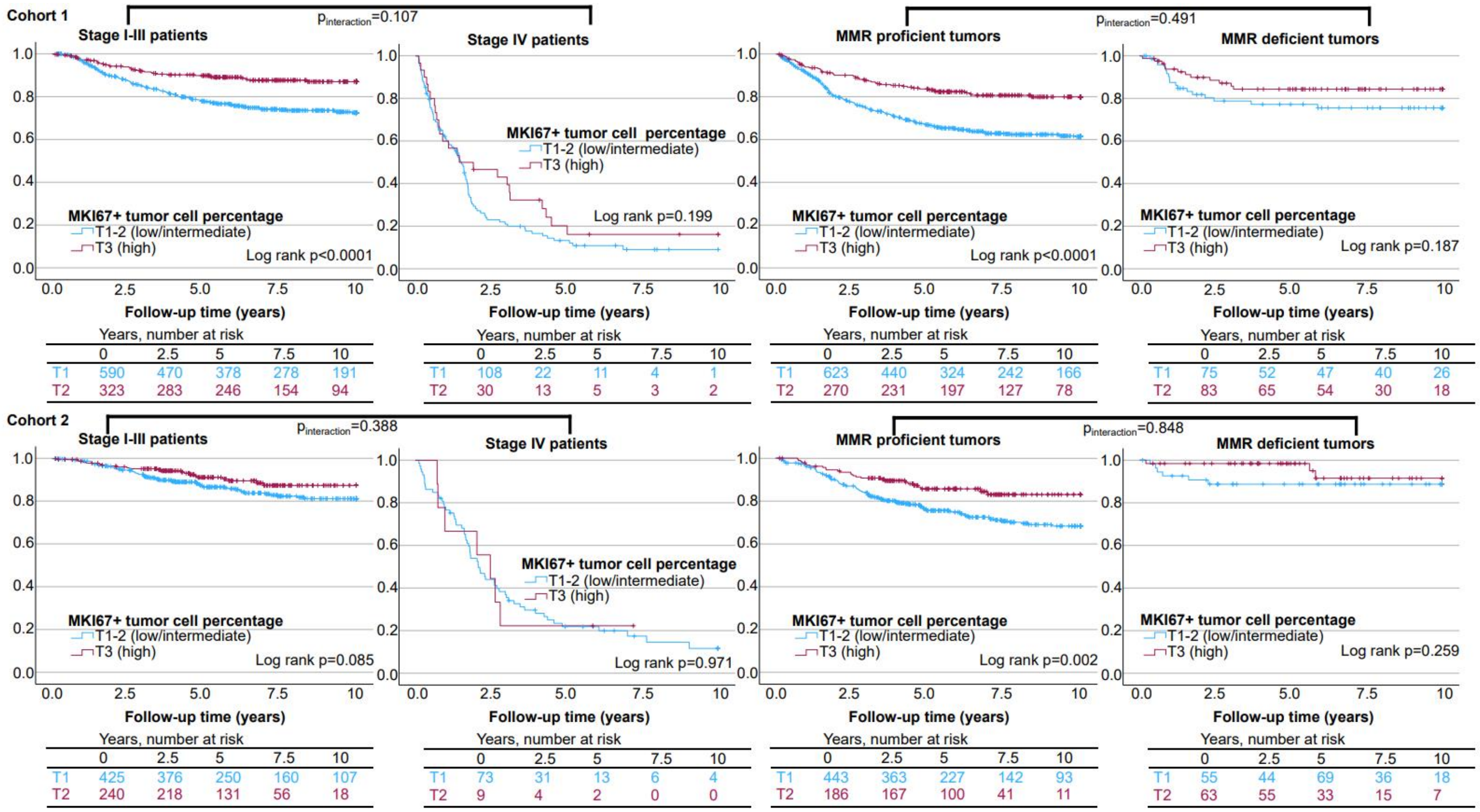

Figure S4. Kaplan-Meier curves for MKI67+ tumor cell percentage categorized by stage (stage I-III, IV) and MMR status (proficient, deficient). $p_{interaction}$ values were calculated using the Wald test for the cross product of MKI67+ tumor cell percentage and stage (I-III vs. IV)/MMR status (proficient vs. deficient) in Cox regression models.
