# Supplementary material for: Unraveling the Effects and Characteristics of Proliferating Tumor and Cytotoxic T Cells in Colorectal Cancer
Source: Clin Cancer Res. 2025 Nov 7;32(2):350–62. doi: 10.1158/1078-0432.CCR-25-2026 (PMC12809117; doi:10.1158/1078-0432.CCR-25-2026)
Supplement: Supplementary Figure S5 — Subgroup analyses of cancer-specific survival according to MKI67+ tumor cell percentage in Cohort 1 and Cohort 2. [file ccr-25-2026_supplementary_figure_s5_suppfs5.pdf]

Kastinen et al. Unraveling the effects and characteristics of proliferating tumor and cytotoxic T cells in colorectal cancer

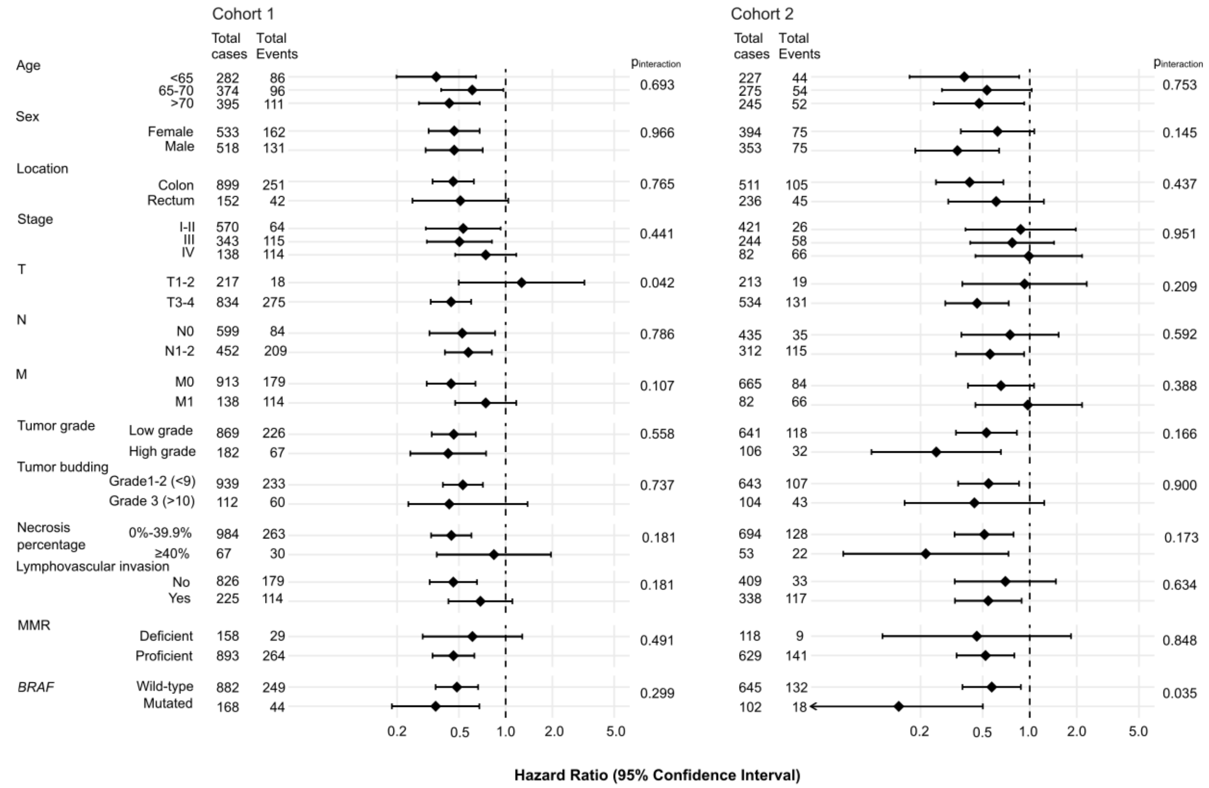

Figure S5. Subgroup analyses of cancer-specific survival according to MKI67+ tumor cell percentage in Cohort 1 and Cohort 2. The forest plots present hazard ratios and 95% confidence intervals for high (vs. low/intermediate) MKI67+ tumor cell percentage. Abbreviations: MMR, mismatch repair.
