# Supplementary material for: Unraveling the Effects and Characteristics of Proliferating Tumor and Cytotoxic T Cells in Colorectal Cancer
Source: Clin Cancer Res. 2025 Nov 7;32(2):350–62. doi: 10.1158/1078-0432.CCR-25-2026 (PMC12809117; doi:10.1158/1078-0432.CCR-25-2026)
Supplement: Supplementary Figure S6 — Kaplan-Meier curves for adjuvant treatment status in relation to MKI67+ tumor cell percentage in Cohort 2 [file ccr-25-2026_supplementary_figure_s6_suppfs6.pdf]

Kastinen et al. Unraveling the effects and characteristics of proliferating tumor and cytotoxic T cells in colorectal cancer

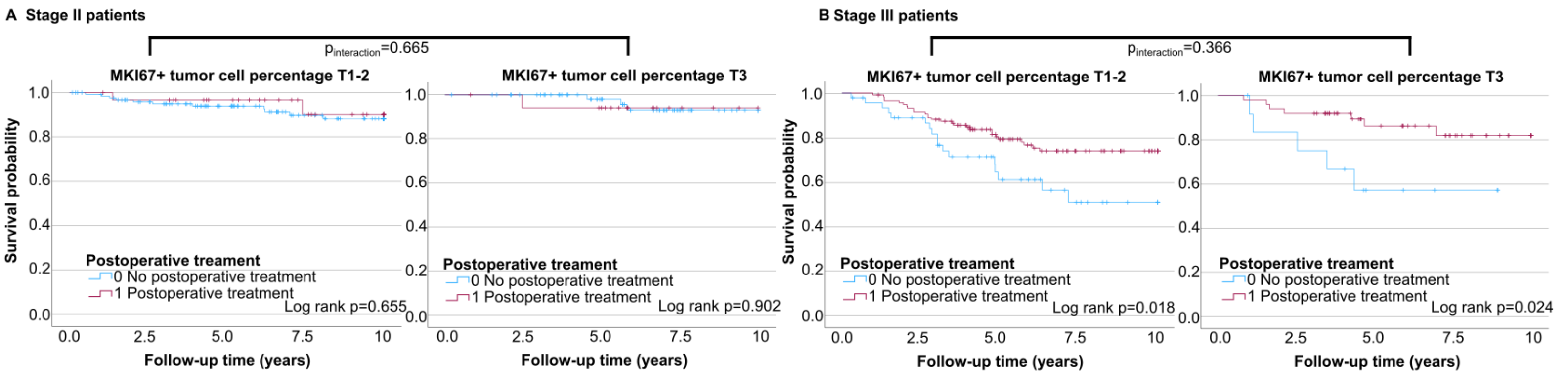

	0	2.5	5	7.5	10
0	126	108	84	57	42
1	31	29	23	14	8

	0	2.5	5	7.5	10
0	67	61	43	19	8
1	18	16	14	4	1

	0	2.5	5	7.5	10
0	47	37	18	8	3
1	119	108	69	44	28

	0	2.5	5	7.5	10
0	14	10	4	2	0
1	50	46	27	15	5

Figure S6. Kaplan-Meier curves for adjuvant treatment status in relation to MKI67+ tumor cell percentage in Cohort 2. **A.** Stage II patients. **B**. Stage III patients. Altogether N=218 received adjuvant treatment (49 in stage II and 169 in stage III). 119 patients receiving oxaliplatin-based therapy (e.g., XELOX or FOLFOX), 84 patients receiving fluoropyrimidine alone, and 15 patients receiving other treatments (e.g., chemoradiotherapy). Patients who died in less than 30 days after surgery were excluded from the analyses. $p_{interaction}$ values were calculated using the Wald test for the cross product of MKI67+ tumor cell percentage (T1-2 vs. T3) and adjuvant treatment status (no vs. yes) in Cox regression models.
