# Supplementary material for: Unraveling the Effects and Characteristics of Proliferating Tumor and Cytotoxic T Cells in Colorectal Cancer
Source: Clin Cancer Res. 2025 Nov 7;32(2):350–62. doi: 10.1158/1078-0432.CCR-25-2026 (PMC12809117; doi:10.1158/1078-0432.CCR-25-2026)
Supplement: Supplementary Figure S7 — Kaplan-Meier curves for proliferating and non-proliferating CD8+ T cell densities categorized by stage (stage I-III, IV) and MMR status (proficient, deficient) in Cohorts 1 and 2. [file ccr-25-2026_supplementary_figure_s7_suppfs7.pdf]

Kastinen et al. Unraveling the effects and characteristics of proliferating tumor and cytotoxic T cells in colorectal cancer

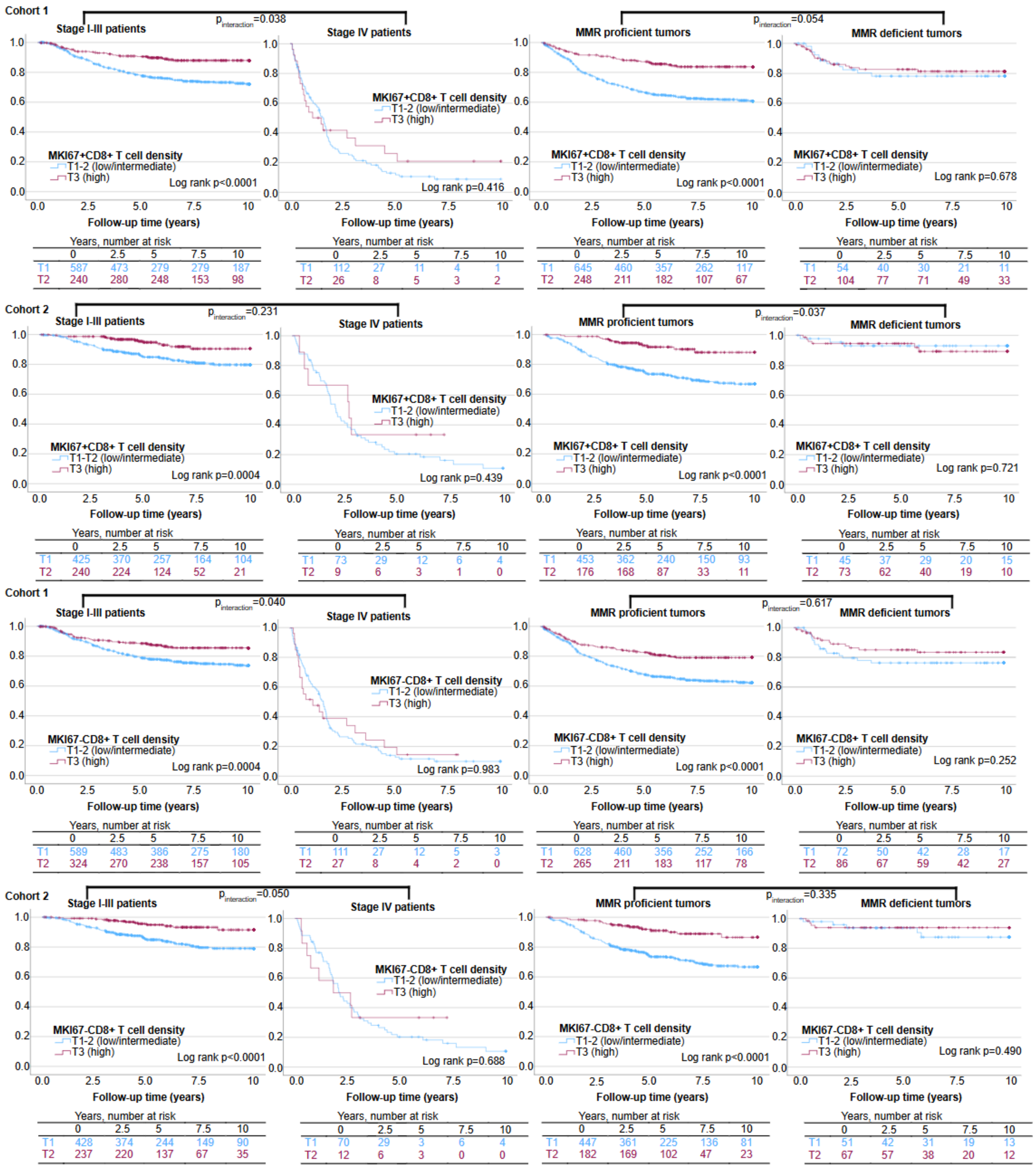

Figure S7. Kaplan-Meier curves for proliferating and non-proliferating CD8+ T cell densities categorized by stage (stage I-III, IV) and MMR status (proficient, deficient) in Cohorts 1 and 2. $p_{interaction}$ values were calculated using the Wald test for the cross product of immune cell density (T1-2 vs. T3) and stage (I-III vs. IV)/MMR status (proficient vs. deficient) in Cox regression models.
