# Supplementary material for: Unraveling the Effects and Characteristics of Proliferating Tumor and Cytotoxic T Cells in Colorectal Cancer
Source: Clin Cancer Res. 2025 Nov 7;32(2):350–62. doi: 10.1158/1078-0432.CCR-25-2026 (PMC12809117; doi:10.1158/1078-0432.CCR-25-2026)
Supplement: Supplementary Figure S8 — Receiver-operating characteristics (ROC) analysis for cancer-specific survival. [file ccr-25-2026_supplementary_figure_s8_suppfs8.pdf]

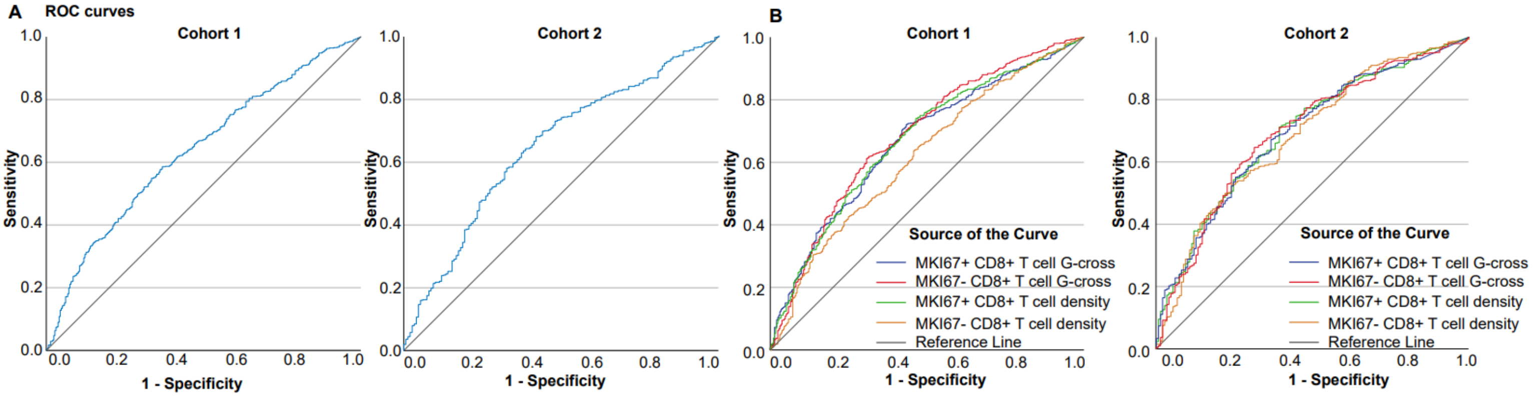

	Cohort 1		Cohort 2	
	Area	CI 95%	Area	CI 95%
MKI67+ tumor cell percentage	0.636	0.60-0.67	0.646	0.60-0.70

	Cohort 1		Cohort 2	
	Area	CI 95%	Area	CI 95%
MKI67+ CD8+ T cell G-cross	0.665	0.63-0.70	0.693	0.65-0.74
MKI67- CD8+ T cell G-cross	0.684	0.65-0.72	0.696	0.65-0.74
MKI67+ CD8+ T cell density	0.669	0.63-0.71	0.696	0.66-0.74
MKI67- CD8+ T cell density	0.619	0.58-0.66	0.685	0.64-0.73

Figure S8. Receiver-operating characteristics (ROC) analysis for cancer-specific survival. **A.** ROC curves for MKI67+ tumor cell percentage. **B**. ROC curves for CD8+ T cell densities and G-cross function values. N=1051 for Cohort 1 and 747 for cohort 2. Patients who died in less than 30 days are excluded from analysis.
