# Supplementary material for: Unraveling the Effects and Characteristics of Proliferating Tumor and Cytotoxic T Cells in Colorectal Cancer
Source: Clin Cancer Res. 2025 Nov 7;32(2):350–62. doi: 10.1158/1078-0432.CCR-25-2026 (PMC12809117; doi:10.1158/1078-0432.CCR-25-2026)
Supplement: Supplementary Table S1 — Representativeness of the study participants. [file ccr-25-2026_supplementary_table_s1_suppts1.pdf]

Kastinen et al. Unraveling the effects and characteristics of proliferating tumor and cytotoxic T cells in colorectal cancer

Table S1. Representativeness of the study participants

Cancer type(s)/subtype(s)/stage(s)/condition<br>Consideration related to:	Colorectal Cancer (CRC)
Sex	CRC is slightly more common in men than in women. (1) It is estimated that men have 8% risk of colorectal cancer compared to women with 7% risk. Incidence rates in the U.S. were 40.7 per 100 000 for men and 30.6 per 100 000 for women between years 2016 and 2020. (2)
Age	The incidence of CRC rises with age. (1) However, incidence rate has begun increasing amongst younger individuals (aged <55 years) by 1-2% per year. (2)
Ethnicity	The incidence and mortality rates of CRC differ in relation to ethnicity. In the U.S., American Indian/Alaska Native (50.0 per 100 000), and Black populations (40.8 per 100 000) experience the highest rates, while Asian American/Pacific Islander populations have the lowest (28.1 per 100 000) (2).
Geography	Incidence rates of CRC are three to four times higher in developed countries compared to developing countries. The highest incidence is observed in Europe, Australia/New Zealand, Northern America, and Eastern Asia, while lowest rates are in Africa and South and Central Asia. (1)
Other Considerations	CRC 5-year survival rate was approximately 64% between years 2013 and 2019 in the United States. (2)
Overall representativeness of the participants of this study	The ethnicity of patients was not recorded; however, they were treated in Finnish hospitals where the majority of the population is non-Hispanic White. To confirm the applicability of these findings to patients of diverse ethnic backgrounds, further research is needed. The female-to-male ratio was 1:1 in Cohort 1 and 1.13:1 in Cohort 2. Female patients were marginally overrepresented in our study cohorts compared to global cancer statistics of CRC. The age distribution of our cohorts aligns with previously reported data.

1. Bray F, Laversanne M, Sung H, Ferlay J, Siegel RL, Soerjomataram I, et al. Global cancer statistics 2022: GLOBOCAN estimates of incidence and mortality worldwide for 36 cancers in 185 countries. CA Cancer J Clin. 2024;74(3):229–63.
2. Siegel, R. L., Giaquinto, A. N., & Jemal, A. (2024). Cancer statistics, 2024. *CA: a cancer journal for clinicians*, *74*(1), 12–49. https://doi.org/10.3322/caac.21820
