# Supplementary material for: Unraveling the Effects and Characteristics of Proliferating Tumor and Cytotoxic T Cells in Colorectal Cancer
Source: Clin Cancer Res. 2025 Nov 7;32(2):350–62. doi: 10.1158/1078-0432.CCR-25-2026 (PMC12809117; doi:10.1158/1078-0432.CCR-25-2026)
Supplement: Supplementary Table S2 — Reagents and resources used in the study methods. [file ccr-25-2026_supplementary_table_s2_suppts2.pdf]

Kastinen et al. Unraveling the effects and characteristics of proliferating tumor and cytotoxic T cells in colorectal cancer

Table S2. Reagents and resources used in the study methods.

Reagents or resource	Source	Identifier
**Antibodies**		
CD8	Leica Biosystems	CD8-4B11-L-U, RRID: AB_3676740
MKI67	Cell Marque	275R-16, RRID: AB_1158037
CK	Leica Biosystems	NCL-L-AE1/AE3-601, RRID: AB_2924990
GZMB	Cell Signaling	46890, RRID: AB_2799313
**Immunohistochemistry reagents**		
Bond Epitope Retrieval Solution 1	Leica Biosystems	AR9961
Bond Epitope Retrieval Solution 2	Leica Biosystems	AR9640
Bond Polymer Refine Detection kit	Leica Biosystems	DS9800
Bond Epitope Retrieval Solution 1	Leica Biosystems	AR9961
Green Chromogen	Leica Biosystems	DC9913
Bond Polymer Refine Red detection kit	Leica Biosystems	DS9390
**Equipment**		
Leica Bond RX research stainer	Leica Biosystems	RRID:SCR_025548
Leica Aperio AT2 scanner	Leica Biosystems	RRID:SCR_021256
**Software**		
R	R Core Team	RRID:SCR_001905
RStudio	RStudio Team	RRID:SCR_000432
SPSS statistics software	IBM SPSS Statistics	RRID:SCR_016479
Qupath	Bankhead P, et al. Sci Rep. 2017;7:16878.	RRID:SCR_018257
