# Supplementary material for: Unraveling the Effects and Characteristics of Proliferating Tumor and Cytotoxic T Cells in Colorectal Cancer
Source: Clin Cancer Res. 2025 Nov 7;32(2):350–62. doi: 10.1158/1078-0432.CCR-25-2026 (PMC12809117; doi:10.1158/1078-0432.CCR-25-2026)
Supplement: Supplementary Table S3 — Multivariable Cox regression analysis for cancer-specific survival according to tumor proliferation rate and covariates in Cohorts 1 and 2. [file ccr-25-2026_supplementary_table_s3_suppts3.pdf]

Table S3. Multivariable Cox regression analysis for cancer-specific survival according to tumor proliferation rate and covariates in Cohorts 1 and 2.

	Cancer-specific survival Multivariable HR (95% CI)	
	Cohort 1	Cohort 2
MKI67+ tumor cell percentage		
Low (T1)	1 (referent)	1 (referent)
Intermediate (T2)	0.94 (0.72-1.23)	0.89 (0.59-1.34)
High (T3)	0.60 (0.43-0.83)	0.92 (0.55-1.52)
Age		
<65	1 (referent)	1 (referent)
65-75	1.13 (0.84-1.53)	1.79 (1.17-2.73)
>75	1.81 (1.35-2.44)	2.75 (1.77-4.28)
Sex		
Male	1 (referent)	1 (referent)
Female	0.83 (0.65-1.06)	0.95 (0.68-1.32)
Year of operation		
2000-2005	1 (referent)	-
2006-2010	0.59 (0.44-0.78)	1 (referent)
2011-2015	0.48 (0.36-0.64)	0.97 (0.63-1.48)
2016-2020	-	0.67 (0.43-1.06)
Tumor location		
Proximal colon	1 (referent)	1 (referent)
Distal colon	0.85 (0.65-1.11)	1.20 (0.78-1.83)
Rectum	0.83 (0.57-1.20)	0.92 (0.60-1.41)
AJCC disease stage		
I-II	1 (referent)	1 (referent)
III	2.91 (2.11-4.01)	2.59 (1.56-4.28)
IV	17.06 (12.05-24.14)	18.32 (10.85-32.00)
Tumor budding		
BD1	1 (referent)	1 (referent)
BD2	1.31 (0.96-1.78)	1.67 (1.07-2.57)
BD3	1.26 (0.92-1.74)	1.87 (1.23-2.84)
Tumor grade		
Low-grade	1 (referent)	1 (referent)
High-grade	1.77 (1.31-2.40)	1.26 (0.82-1.93)
Lymphovascular invasion		
No	1 (referent)	1 (referent)
Yes	1.73 (1.33-2.24)	1.91 (1.23-2.98)
MMR status		
MMR proficient	1 (referent)	1 (referent)
MMR deficient	0.58 (0.34-0.96)	0.55 (0.27-1.19)
BRAF mutation		
Wild-type	1 (referent)	1 (referent)
Mutant	1.48 (0.97-2.28)	1.47 (0.78-2.74)
