# Supplementary material for: Unraveling the Effects and Characteristics of Proliferating Tumor and Cytotoxic T Cells in Colorectal Cancer
Source: Clin Cancer Res. 2025 Nov 7;32(2):350–62. doi: 10.1158/1078-0432.CCR-25-2026 (PMC12809117; doi:10.1158/1078-0432.CCR-25-2026)
Supplement: Supplementary Table S4 — Sensitivity analysis of Cox regression models for cancer-specific survival according to tumor cell proliferation and proliferating and non-proliferating CD8+ T cells, using alternative cut-points. [file ccr-25-2026_supplementary_table_s4_suppts4.pdf]

Table S4. Sensitivity analysis of Cox regression models for cancer-specific survival according to tumor cell proliferation and proliferating and non-proliferating CD8+ T cells, using alternative cut-points.

	**Cohort 1**				**Cohort 2**			
	No. Of cases	No. Of events	Univariable HR (95% CI)	Multivariable HR (95% CI)	No. Of cases	No. Of events	Univariable HR (95% CI)	Multivariable HR (95% CI)
**MKI67+ Tumor cell percentage**								
Low	525	190	1 (referent)	1 (referent)	373	102	1 (referent)	1 (referent)
High	526	103	0.49 (0.39-0.63)	0.73 (0.56-0.95)	374	48	0.47 (0.33-0.66)	1.02 (0.69-1.51)
p value			<0.0001	0.019			<0.0001	0.917
**MKI67+ CD8+ T cells**								
Low	524	200	1 (referent)	1 (referent)	373	108	1 (referent)	1 (referent)
High	527	93	0.42 (0.33-0.54)	0.64 (0.48-0.85)	374	42	0.38 (0.27-0.55)	0.75 (0.49-1.15)
p value			<0.0001	0.002			<0.0001	0.184
**MKI67– CD8+ T cells**								
Low	524	176	1 (referent)	1 (referent)	374	107	1 (referent)	1 (referent)
High	527	117	0.62 (0.49-0.78)	0.85 (0.67-1.09)	373	43	0.39 (0.27-0.55)	0.61 (0.40-0.91)
p value			<0.0001	0.206			<0.0001	0.015

Variables were dichotomized by the median.

Multivariable Cox regression models were adjusted for age (<65, 65-75, >75), sex (female, male), stage (I-II, III, IV), lymphovascular invasion (no, yes), grade (low-grade, high-grade), tumor budding (grade I,II, III), year of operation (Cohort 1: 2000-2005, 2006-2010, 2011-2015; Cohort 2: 2006-2010, 2011-2015, 2016-2020), tumor location (proximal colon, distal colon, rectum), *BRAF* status (wild-type, mutant), and mismatch repair status (proficient, deficient).
