# Supplementary material for: Unraveling the Effects and Characteristics of Proliferating Tumor and Cytotoxic T Cells in Colorectal Cancer
Source: Clin Cancer Res. 2025 Nov 7;32(2):350–62. doi: 10.1158/1078-0432.CCR-25-2026 (PMC12809117; doi:10.1158/1078-0432.CCR-25-2026)
Supplement: Supplementary Table S5 — Cox regression models for cancer-specific survival according to tumor proliferation rate in the invasive margin (im) and tumor center (ct) [file ccr-25-2026_supplementary_table_s5_suppts5.pdf]

Kastinen et al. Unraveling the effects and characteristics of proliferating tumor and cytotoxic T cells in colorectal cancer

Table S5. Cox regression models for cancer-specific survival according to tumor proliferation rate in the invasive margin (im) and tumor center (ct)

	Cohort 1				Cohort 2			
	No. Of cases	No. Of events	Univariable HR (95% CI)	Multivariable HR (95% CI)	No. Of cases	No. Of events	Univariable HR (95% CI)	Multivariable HR (95% CI)
**MKI67+ tumor cell percentage im**								
T1	329	125	1 (referent)	1 (referent)	245	76	1 (referent)	1 (referent)
T2	330	95	0.75 (0.58-0.98)	0.98 (0.74-1.29)	244	40	0.53 (0.36-0.78)	0.92 (0.61-1.39)
T3	329	60	0.44 (0.32-0.60)	0.68 (0.48-0.95)	245	30	0.40 (0.26-0.60)	0.97 (0.59-1.59)
p_{trend}			<0.001	0.036			<0.001	0.836
**MKI67+ tumor cell percentage ct**								
T1	347	121	1 (referent)	1 (referent)	246	76	1 (referent)	1 (referent)
T2	350	110	0.90 (0.69-1.16)	1.14 (0.87-1.48)	247	46	0.61 (0.42-0.88)	0.90 (0.59-1.36)
T3	346	59	0.44 (0.32-0.60)	0.68 (0.49-0.96)	246	27	0.34 (0.22-0.52)	0.79 (0.47-1.31)
p_{trend}			<0.001	0.062			<0.001	0.348

Multivariable Cox regression models were adjusted for age (<65, 65-75, >75), sex (female, male), stage (I-II, III, IV), lymphovascular invasion (no, yes), grade (low-grade, high-grade), tumor budding (grade I,II, III), year of operation (Cohort 1: 2000-2005, 2006-2010, 2011-2015; Cohort 2: 2006-2010, 2011-2015, 2016-2020), tumor location (proximal colon, distal colon, rectum), *BRAF* status (wild-type, mutant) and mismatch repair status (proficient, deficient).

p_{trend} values were calculated by using the three ordinal categories of immune cell densities as continuous variables in univariable and multivariable Cox proportional hazard regression models.
