# Supplementary material for: Unraveling the Effects and Characteristics of Proliferating Tumor and Cytotoxic T Cells in Colorectal Cancer
Source: Clin Cancer Res. 2025 Nov 7;32(2):350–62. doi: 10.1158/1078-0432.CCR-25-2026 (PMC12809117; doi:10.1158/1078-0432.CCR-25-2026)
Supplement: Supplementary Table S6 — Cox regression models for cancer-specific survival according to tumor cell proliferation and proliferating and non-proliferating CD8+ T cells, stratified by stage (I-III vs. IV) [file ccr-25-2026_supplementary_table_s6_suppts6.pdf]

Table S6. Cox regression models for cancer-specific survival according to tumor cell proliferation and proliferating and non-proliferating CD8+ T cells, stratified by stage (I-III vs. IV)

	**Cohort 1**				**Cohort 2**			
	No. Of cases	No. Of events	Univariable HR (95% CI)	Multivariable HR (95% CI)	No. Of cases	No. Of events	Univariable HR (95% CI)	Multivariable HR (95% CI)
**Stage I-III tumors**								
**MKI67+ tumor cell percentage**								
T1-2	590	142	1 (referent)	1 (referent)	425	62	1 (referent)	1 (referent)
T3	323	37	0.45 (0.31-0.64)	0.57 (0.39-0.84)	240	22	0.65 (0.40-1.07)	1.00 (0.59-0.1.69)
p value			<0.0001	0.004			0.088	0.992
**Stage IV tumors**								
**MKI67+ tumor cell percentage**								
T1-2	108	90	1 (referent)	1 (referent)	73	59	1 (referent)	1 (referent)
T3	30	24	0.74 (0.47-1.17)	0.60 (0.35-1.01)	9	7	0.99 (0.45-2.16)	1.36 (0.54-3.40)
p value			0.201	0.055			0.971	0.512
$p_{interaction}$			0.107	0.054			0.388	0.895
**Stage I-III tumors**								
**MKI67+ CD8+ T cells**								
T1-2	587	144	1 (referent)	1 (referent)	425	70	1 (referent)	1 (referent)
T3	326	35	0.41 (0.29-0.60)	0.49 (0.33-0.74)	240	14	0.37 (0.21-0.66)	0.49 (0.27-0.91)
p value			<0.0001	0.0005			0.0007	0.023
**Stage IV tumors**								
**MKI67+ CD8+ T cells**								
T1-2	112	95	1 (referent)	1 (referent)	73	60	1 (referent)	1 (referent)
T3	26	19	0.82 (0.50-1.34)	0.90 (0.50-1.61)	9	6	0.72 (0.31-1.67)	0.50 (0.17-1.47)
p value			0.417	0.723			0.441	0.209
$p_{interaction}$			0.038	0.012			0.231	0.617
**Stage I-III tumors**								
**MKI67– CD8+ T cells**								
T1-2	589	137	1 (referent)	1 (referent)	425	70	1 (referent)	1 (referent)
T3	324	42	0.54 (0.38-0.76)	0.59 (0.41-0.84)	240	14	0.33 (0.18-0.58)	0.43 (0.24-0.79)
p value			0.0005	0.004			0.0002	0.007
**Stage IV tumors**								
**MKI67– CD8+ T cells**								
T1-2	111	93	1 (referent)	1 (referent)	70	58	1 (referent)	1 (referent)
T3	27	21	1.0 (0.62-1.60)	1.33 (0.80-2.20)	12	8	0.86 (0.41-1.80)	0.76 (0.29-1.98)
p value			0.983	0.273			0.688	0.568
$p_{interaction}$			0.040	0.003			0.050	0.254

Multivariable Cox regression models were adjusted for age (<65, 65-75, >75), sex (female, male), stage (I, II, III; only in stage I-III category), lymphovascular invasion (no, yes), grade (low-grade, high-grade), tumor budding (grade I,II, III), year of operation (Cohort 1: 2000-2005, 2006-2010, 2011-2015; Cohort 2: 2006-2010, 2011-2015, 2016-2020), tumor location (proximal colon, distal colon, rectum), *BRAF* status (wild-type, mutant), and mismatch repair status (proficient, deficient).

$p_{interaction}$ values were calculated using the Wald test for the cross product of tumor MKI67+ percentage/immune cell density (T1-2 vs. T3) and stage (I-III vs. IV) in Cox regression models.
