# Supplementary material for: Unraveling the Effects and Characteristics of Proliferating Tumor and Cytotoxic T Cells in Colorectal Cancer
Source: Clin Cancer Res. 2025 Nov 7;32(2):350–62. doi: 10.1158/1078-0432.CCR-25-2026 (PMC12809117; doi:10.1158/1078-0432.CCR-25-2026)
Supplement: Supplementary Table S7 — Cox regression models for cancer-specific survival according to tumor cell proliferation and proliferating and non-proliferating CD8+ T cells, stratified by mismatch repair status. [file ccr-25-2026_supplementary_table_s7_suppts7.pdf]

Table S7. Cox regression models for cancer-specific survival according to tumor cell proliferation and proliferating and non-proliferating CD8+ T cells, stratified by mismatch repair status.

	Cohort 1				Cohort 2			
	No. Of cases	No. Of events	Univariable HR (95% CI)	Multivariable HR (95% CI)	No. Of cases	No. Of events	Univariable HR (95% CI)	Multivariable HR (95% CI)
**MMR proficient**								
**Tumor MKI67+ percentage**								
T1-2	623	215	1 (referent)	1 (referent)	443	115	1 (referent)	1 (referent)
T3	270	49	0.46 (0.34-0.63)	0.60 (0.44-0.84)	186	26	0.52 (0.34-0.80)	1.02 (0.63-1.64)
p value			<0.0001	0.002			0.003	0.939
**MMR deficient***								
**Tumor MKI67+ percentage**								
T1-2	75	17	1 (referent)	1 (referent)	55	6	1 (referent)	
T3	83	12	0.61 (0.29-1.28)	0.75 (0.30-1.90)	63	3	0.46 (0.11-1.84)	
p value			0.192	0.543			0.271	
$p_{interaction}$			0.491	0.587			0.848	
**MMR proficient**								
**MKI67+ CD8+ T cells**								
T1-2	645	228	1 (referent)	1 (referent)	453	127	1 (referent)	1 (referent)
T3	248	36	0.36 (0.26-0.52)	0.51 (0.36-0.74)	176	14	0.27 (0.16-0.47)	0.38 (0.21-0.69)
p value			<0.0001	0.0004			<0.0001	0.002
**MMR deficient***								
**MKI67+ CD8+ T cells**								
T1-2	54	11	1 (referent)	1 (referent)	45	3	1 (referent)	
T3	104	18	0.85 (0.40-1.81)	0.91 (0.39-2.14)	73	6	1.28 (0.32-5.16)	
p value			0.678	0.829			0.721	
$p_{interaction}$			0.054	0.399			0.037	
**MMR proficient**								
**MKI67– CD8+ T cells**								
T1-2	628	214	1 (referent)	1 (referent)	447	124	1 (referent)	1 (referent)
T3	265	50	0.52 (0.38-0.70)	0.73 (0.53-1.01)	182	17	0.31 (0.19-0.52)	0.46 (0.27-0.78)
p value			<0.0001	0.054			<0.0001	0.004
**MMR deficient***								
**MKI67– CD8+ T cells**								
T1-2	72	16	1 (referent)	1 (referent)	51	5	1 (referent)	
T3	86	13	0.65 (0.32-1.36)	0.65 (0.28-1.50)	67	4	0.63 (0.17-2.35)	
p value			0.256	0.315			0.494	
$p_{interaction}$			0.617	0.736			0.335	

Multivariable Cox regression models were adjusted for age (<65, 65-75, >75), sex (female, male), stage (I-II, III, IV), lymphovascular invasion (no, yes), grade (low-grade, high-grade), tumor budding (grade I,II, III), year of operation (Cohort 1: 2000-2005, 2006-2010, 2011-2015; Cohort 2: 2006-2010, 2011-2015, 2016-2020), tumor location (proximal colon, distal colon, rectum), and *BRAF* status (wild-type, mutant).

*In Cohort 2, the MMR deficient subgroup had too few endpoints to permit reliable multivariable analysis.

$p_{interaction}$ values were calculated using the Wald test for the cross product of tumor MKI67+ percentage/immune cell density (T1-2 vs. T3) and MMR status (proficient vs. deficient) in Cox regression models.
