# Supplementary material for: Unraveling the Effects and Characteristics of Proliferating Tumor and Cytotoxic T Cells in Colorectal Cancer
Source: Clin Cancer Res. 2025 Nov 7;32(2):350–62. doi: 10.1158/1078-0432.CCR-25-2026 (PMC12809117; doi:10.1158/1078-0432.CCR-25-2026)
Supplement: Supplementary Table S8 — Tumor and patient characteristics in relation to proliferating and non-proliferating cytotoxic T cell densities in Cohorts 1 and 2. [file ccr-25-2026_supplementary_table_s8_suppts8.pdf]

Table S8. Tumor and patient characteristics in relation to proliferating and non-proliferating cytotoxic T cell densities in Cohorts 1 and 2.

	Cohort 1 Total N 1088	Median (IQR) MKI67+CD8+ T cell density, 1/mm^2	MKI67- CD8+ T cell density, 1/mm^2	Cohort 2 Total N 752	Median (IQR) MKI67+CD8+ T cell density, 1/mm^2	MKI67- CD8+ T cell density, 1/mm^2
**Sex**						
Male	551 (51%)	18.5 (6.84-44.6)	138 (86.2-256)	398 (53%)	28 (7.6-73)	160 (78-294)
Female	537 (49%)	19.1 (6.68-53.2)	152 (77.4-281)	354 (47%)	33 (8.0-79)	153 (77-313)
**p value**		0.451	0.411		0.545	0.687
**Age**						
<65	285 (26%)	17 (5.5-43)	140 (78-251)	227 (30%)	25 (4.5-60)	131 (54-269)
65-75	379 (35%)	22 (7.4-48)	149 (89-270)	276 (37%)	30 (9.1-81)	159 (89.46-307)
>75	424 (39%)	19 (6.7-53)	145 (82-285)	249 (34%)	34 (9.2-81)	187 (101-321)
**p value**		0.149	0.421		0.247	0.004
**Tumor location**						
Proximal colon	530 (49%)	25 (7.8-63)	163 (92-300)	314 (42%)	36 (9.2-93)	183 (94-324)
Distal colon	403 (37%)	14 (5.8-36)	126 (74-239)	201 (27%)	25 (6.8-62)	136 (60-271)
Rectum	155 (14%)	18 (7.1-47)	151 (82-263)	237 (32%)	28 (7.0-62)	160. (81-313)
**p value**		<0.0001	<0.0001		0.008	0.009
**Stage**						
I	180 (17%)	27 (10-60)	169 (92-287)	173 (23%)	48 (14-118)	211 (104-362)
II	406 (37%)	24 (7.4-61)	158 (90-295)	249 (33%)	33 (10-80)	175 (98-318)
III	352 (32%)	16 (6.2-41)	139 (80-261)	247 (33%)	27 (7.6-60)	149 (76-269)
IV	150 (14%)	9 (3.7-26)	111 (69-193)	83 (11%)	7.6 (1.7-23)	75 (29-155)
**p value**		<0.0001	0.0001		<0.0001	<0.0001
**WHO grade**						
Low-grade	895 (82%)	17 (6.4-43)	139 (82-255)	644 (86%)	28 (7.1-68)	152 (75-136)
High-grade	193 (18%)	30 (8.4-117)	184 (87-416)	108 (14%)	41 (13-136)	200 (99-389)
**p value**		<0.0001	<0.0001		0.001	0.005
**Lymphovascular invasion**						
No	847 (78%)	23 (7.9-56)	150 (85-281)	411 (55%)	39 (10-85)	194 (97-336)
Yes	241 (22%)	10 (4.9-28)	128 (76-229)	341 (45%)	20 (5.3-54)	138 (66-250)
**p value**		<0.0001	0.015		<0.0001	<0.0001
**Tumor necrosis percentage**						
<3%	249 (23%)	27 (9.1-66)	168 (92-337)	222 (30%)	43 (11-86)	184 (97-310)
3-39.9%	768 (71%)	18 (6.2-45)	140 (81-258)	477 (63%)	25 (6.6-65)	148 (67-290)
≥40%	71 (6.5%)	17 (4.8-39)	136 (69-257)	53 (7.0%)	33 (4.9-116)	202 (85-387)
**p value**		0.0004	0.002		0.001	0.015
**MMR status**						
MMR proficient	924 (85%)	16 (6.0-39)	136 (79-251)	633 (84%)	25 (6.8-59)	145 (71-272)
MMR deficient	164 (15%)	56 (20-153)	241 (126-487)	119 (16%)	84 (35-206)	280 (158-526)
**p value**		<0.0001	<0.0001		<0.0001	<0.0001
BRAF* status						
Wild-type	907 (83%)	16 (6.0-42)	136 (79-255)	648 (86%)	26 (6.8-63)	150 (74-282)
Mutant	179 (16%)	40 (16-107)	207 (118-394)	104 (14%)	79 (27-153)	245 (140-437)
**p value**		<0.0001	<0.0001		<0.0001	<0.0001
**P53 immunohistochemistry****						
Mutated pattern	579 (53%)	15 (6.2-40)	134 (80-247)	432 (57%)	30 (7.9-72)	151 (75-309)
Wild type pattern	508 (47%)	25 (7.5-63)	164 (87-299)	320 (43%)	31 (8.1-78)	166 (83-295)
**p value**		<0.0001	0.0004		0.744	0.406

*Data missing from 2 patients in Cohort 1

**Data missing from 1 patient in Cohort 1
