# Supplementary material for: Unraveling the Effects and Characteristics of Proliferating Tumor and Cytotoxic T Cells in Colorectal Cancer
Source: Clin Cancer Res. 2025 Nov 7;32(2):350–62. doi: 10.1158/1078-0432.CCR-25-2026 (PMC12809117; doi:10.1158/1078-0432.CCR-25-2026)
Supplement: Supplementary Table S9 — Multivariable Cox regression analysis for cancer-specific survival according to non-proliferating cytotoxic T cell densities and covariates in Cohorts 1 and 2. [file ccr-25-2026_supplementary_table_s9_suppts9.pdf]

Table S9. Multivariable Cox regression analysis for cancer-specific survival according to non-proliferating cytotoxic T cell densities and covariates in Cohorts 1 and 2.

	Cancer-specific survival Multivariable HR (95% CI)	
	Cohort 1	Cohort 2
MKI67-CD8+ T cell density		
Low (T1)	1 (referent)	1 (referent)
Intermediate (T2)	0.74 (0.57-0.97)	0.89 (0.60-1.32)
High (T3)	0.63 (0.46-0.86)	0.44 (0.26-0.76)
Age		
<65	1 (referent)	1 (referent)
65-75	1.13 (0.84-1.52)	1.92 (1.26-2.95)
>75	1.78 (1.33-2.40)	3.05 (1.95-4.80)
Sex		
Male	1 (referent)	1 (referent)
Female	0.85 (0.67-1.09)	0.94 (0.67-1.31)
Year of operation		
2000-2005	1 (referent)	-
2006-2010	0.61 (0.46-0.81)	1 (referent)
2011-2015	0.49 (0.37-0.66)	0.98 (0.65-1.50)
2016-2020	-	0.76 (0.49-1.17)
Tumor location		
Proximal colon	1 (referent)	1 (referent)
Distal colon	0.85 (0.65-1.11)	1.16 (0.76-1.77)
Rectum	0.84 (0.58-1.21)	0.92 (0.60-1.41)
AJCC disease stage		
I-II	1 (referent)	1 (referent)
III	2.93 (2.13-4.02)	2.56 (1.55-4.24)
IV	16.46 (11.64-23.28)	17.87 (10.38-30.78)
Tumor budding		
BD1	1 (referent)	1 (referent)
BD2	1.37 (1.01-1.87)	1.65 (1.07-2.56)
BD3	1.39 (1.01-1.90)	1.88 (1.25-2.84)
Tumor grade		
Low-grade	1 (referent)	1 (referent)
High-grade	1.85 (1.37-2.50)	1.28 (0.84-1.96)
Lymphovascular invasion		
No	1 (referent)	1 (referent)
Yes	1.77 (1.37-2.28)	1.85 (1.18-2.89)
MMR status		
MMR proficient	1 (referent)	1 (referent)
MMR deficient	0.62 (0.37-1.04)	0.61 (0.28-1.33)
BRAF mutation		
Wild-type	1 (referent)	1 (referent)
Mutant	1.34 (0.88-2.05)	1.76 (0.95-3.26)
