# Supplementary material for: Unraveling the Effects and Characteristics of Proliferating Tumor and Cytotoxic T Cells in Colorectal Cancer
Source: Clin Cancer Res. 2025 Nov 7;32(2):350–62. doi: 10.1158/1078-0432.CCR-25-2026 (PMC12809117; doi:10.1158/1078-0432.CCR-25-2026)
Supplement: Supplementary Table S10 — Multivariable Cox regression analysis for cancer-specific survival according to proliferating cytotoxic T cell densities and covariates in Cohorts 1 and 2. [file ccr-25-2026_supplementary_table_s10_suppts10.pdf]

Table S10. Multivariable Cox regression analysis for cancer-specific survival according to proliferating cytotoxic T cell densities and covariates in Cohorts 1 and 2.

	Cancer-specific survival Multivariable HR (95% CI)	
	Cohort 1	Cohort 2
MKI67+ CD8+ T cell density		
Low (T1)	1 (referent)	1 (referent)
Intermediate (T2)	0.72 (0.55-0.95)	0.75 (0.50-1.13)
High (T3)	0.49 (0.35-0.70)	0.38 (0.21-0.68)
Age		
<65	1 (referent)	1 (referent)
65-75	1.14 (0.84-1.53)	1.90 (1.25-2.90)
>75	1.81 (1.35-2.43)	3.00 (1.92-4.69)
Sex		
Male	1 (referent)	1 (referent)
Female	0.85 (0.67-1.08)	0.91 (0.65-1.28)
Year of operation		
2000-2005	1 (referent)	-
2006-2010	0.62 (0.46-0.82)	1 (referent)
2011-2015	0.52 (0.38-0.69)	1.01 (0.66-1.54)
2016-2020	-	0.85 (0.54-1.33)
Tumor location		
Proximal colon	1 (referent)	1 (referent)
Distal colon	0.84 (0.64-1.10)	1.24 (0.81-1.90)
Rectum	0.83 (0.58-1.20)	0.91 (0.60-1.40)
AJCC disease stage		
I-II	1 (referent)	1 (referent)
III	2.85 (2.07-3.92)	2.60 (1.57-4.29)
IV	15.48 (10.92-21.93)	17.29 (10.05-29.76)
Tumor budding		
BD1	1 (referent)	1 (referent)
BD2	1.32 (0.97-1.80)	1.65 (1.06-2.55)
BD3	1.27 (0.92-1.75)	1.88 (1.24-2.83)
Tumor grade		
Low-grade	1 (referent)	1 (referent)
High-grade	1.83 (1.35-2.47)	1.34 (0.88-2.05)
Lymphovascular invasion		
No	1 (referent)	1 (referent)
Yes	1.73 (1.33-2.23)	1.86 (1.19-2.90)
MMR status		
MMR proficient	1 (referent)	1 (referent)
MMR deficient	0.64 (0.38-1.07)	0.63 (0.29-1.37)
BRAF mutation		
Wild-type	1 (referent)	1 (referent)
Mutant	1.49 (0.97-2.29)	2.02 (1.07-3.80)
