# Supplementary material for: Unraveling the Effects and Characteristics of Proliferating Tumor and Cytotoxic T Cells in Colorectal Cancer
Source: Clin Cancer Res. 2025 Nov 7;32(2):350–62. doi: 10.1158/1078-0432.CCR-25-2026 (PMC12809117; doi:10.1158/1078-0432.CCR-25-2026)
Supplement: Supplementary Table S11 — Cox regression models comparing the prognostic value of CD8+ T cell densities and overall CD8+ T cell densities in Cohorts 1 and 2. [file ccr-25-2026_supplementary_table_s11_suppts11.pdf]

Table S11. Cox regression models comparing the prognostic value of CD8+ T cell densities and overall CD8+ T cell densities in Cohorts 1 and 2.

	No. Of cases	No. Of events	Model 1 (univariable) HR (CI95%)	Model 2 (multivariable) HR (CI95%)	Model 3 (multivariable) HR (CI95%)
**Cohort 1**					
**CD8+ T cells overall density**					
T1	349	135	1 (referent)	1 (referent)	1 (referent)
T2	349	97	0.68 (0.53-0.89)	1.09 (0.81-1.48)	0.92 (0.68-1.26)
T3	353	61	0.40 (0.30-0.54)	1.03 (0.65-1.63)	0.87 (0.54-1.39)
p trend			<0.001	0.786	0.930
**MKI67+CD8+ T cells density**					
T1	347	153	1 (referent)	1 (referent)	1 (referent)
T2	352	86	0.50 (0.39-0.66)	0.49 (0.36-0.67)	0.76 (0.55-1.05)
T3	352	54	0.30 (0.22-0.41)	0.29 (0.18-0.47)	0.55 (0.33-0.91)
p trend			<0.001	<0.001	0.017
**Cohort 2**					
**CD8+ T cells overall density**					
T1	249	80	1 (referent)	1 (referent)	1 (referent)
T2	251	48	0.59 (0.41-0.84)	0.90 (0.57-1.41)	1.12 (0.67-1.87)
T3	248	22	0.26 (0.16-0.42)	0.62 (0.31-1.26)	1.09 (0.49-2.42)
p trend			<0.001	0.232	0.760
**MKI67+CD8+ T cells density**					
T1	249	85	1 (referent)	1 (referent)	1 (referent)
T2	249	45	0.52 (0.36-0.75)	0.59 (0.37-0.93)	0.70 (0.41-1,19)
T3	249	20	0.23 (0.14-0.37)	0.33 (0.16-0.67)	0.35 (0.15-0.83)
p trend			<0.001	0.002	0.021

Model 1: Univariable cox regression analysis. Model 2: Overall CD8+ T cell density and MKI67+CD8 T cell density in the same model. Model 3: Same as Model 2 but with additionally adjusted for age (<65, 65-75, >75), sex (female, male), stage (I-II, III, IV), lymphovascular invasion (no, yes), grade (low-grade, high-grade), tumor budding (I, II and III), year of operation (Cohort 1: 2000-2005, 2006-2010, 2011-2015; Cohort 2: 2006-2010, 2011-2015, 2016-2020), tumor location (proximal colon, distal colon, rectum), *BRAF* status (wild-type, mutant) and mismatch repair status (proficient, deficient). Patients who died in less than 30 days after surgery were excluded from analysis.

p_{trend} values were calculated by using the three ordinal categories of immune cell densities as continuous variables in univariable and multivariable Cox proportional hazard regression models.
