# Supplementary material for: Unraveling the Effects and Characteristics of Proliferating Tumor and Cytotoxic T Cells in Colorectal Cancer
Source: Clin Cancer Res. 2025 Nov 7;32(2):350–62. doi: 10.1158/1078-0432.CCR-25-2026 (PMC12809117; doi:10.1158/1078-0432.CCR-25-2026)
Supplement: Supplementary Table S12 — Cox regression models comparing the prognostic value of CD8+ T cell densities and G-cross function values in Cohorts 1 and 2. [file ccr-25-2026_supplementary_table_s12_suppts12.pdf]

Table S12. Cox regression models comparing the prognostic value of CD8+ T cell densities and G-cross function values in Cohorts 1 and 2.

	No. Of cases	No. Of events	Model 1 (univariable) HR (CI95%)	Model 2 (multivariable) HR (CI95%)	Model 3 (multivariable) HR (CI95%)
**MKI67-CD8+ T cells**					
**Cohort 1**					
**G-cross**					
T1	344	149	1 (referent)	1 (referent)	1 (referent)
T2	351	93	0.55 (0.42-0.71)	0.52 (0.39-0.70)	0.87 (0.64-1.18)
T3	356	51	0.27 (0.20-0.37)	0.24 (0.16-0.37)	0.48 (0.30-0.77)
p trend			<0.001	<0.001	0.007
**Density**					
T1	348	130	1 (referent)	1 (referent)	1 (referent)
T2	352	100	0.72 (0.55-0.93)	1.10 (0.83-1.47)	0.84 (0.63-1.13)
T3	351	63	0.44 (0.32-0.59)	1.17 (0.78-1.75)	1.00 (0.65-1.54)
p trend			<0.001	0.424	0.713
**Cohort 2**					
**G-cross**					
T1	248	86	1 (referent)	1 (referent)	1 (referent)
T2	250	41	0.44 (0.30-0.63)	0.52 (0.33-0.80)	0.63 (0.40-1.00)
T3	249	23	0.24 (0.15-0.39)	0.42 (0.21-0.82)	0.60 (0.30-1.20)
p trend			<0.001	0.002	0.068
**Density**					
T1	249	81	1 (referent)	1 (referent)	1 (referent)
T2	249	48	0.58 (0.41-0.83)	0.87 (0.57-1.33)	1.15 (0.73-1.82)
T3	249	21	0.24 (0.15-0.39)	0.48 (0.24-0.95)	0.63 (0.31-1.29)
p trend			<0.001	0.064	0.392
**MKI67+CD8+ T cells**					
**Cohort 1**					
**G-cross**					
T1	346	150	1 (referent)	1 (referent)	1 (referent)
T2	350	91	0.53 (0.41-0.69)	0.69 (0.49-0.97)	1.02 (0.71-1.46)
T3	355	52	0.28 (0.21-0.39)	0.43 (0.24-0.79)	0.73 (0.39-1.37)
p trend			<0.001	0.004	0.512
**Density**					
T1	347	153	1 (referent)	1 (referent)	1 (referent)
T2	352	86	0.50 (0.39-0.66)	0.67 (0.48-0.95)	0.73 (0.51-1.05)
T3	352	54	0.30 (0.22-0.41)	0.61 (0.34-1.11)	0.63 (0.37-1.16)
p trend			<0.001	0.031	0.075
**Cohort 2**					
**G-cross**					
T1	249	83	1 (referent)	1 (referent)	1 (referent)
T2	249	44	0.53 (0.36-0.76)	0.82 (0.48-1.38)	1.11 (0.64-1.93)
T3	249	23	0.27 (0.17-0.43)	0.76 (0.34-1.71)	1.29 (0.53-3.11)
p trend			<0.001	0.448	0.577
**Density**					
T1	249	85	1 (referent)	1 (referent)	1 (referent)
T2	249	45	0.52 (0.36-0.75)	0.61 (0.36-1.04)	0.69 (0.39-1.21)
T3	249	20	0.23 (0.14-0.37)	0.29 (0.12-0.67)	0.31 (0.12-0.78)
p trend			<0.001	0.004	0.018

Model 2: Cox proportional hazards regression model included the overall density of CD8+ cells and g-cross function values from 20 µm. Model 3: Cox proportional hazards regression model that was based on Model 2 and additionally adjusted for age (<65, 65-75, >75), sex (female, male), stage (I-II, III, IV), lymphovascular invasion (no, yes), grade (low-grade, high-grade), tumor budding (I, II and III), year of operation (Cohort 1: 2000-2005, 2006-2010, 2011-2015; Cohort 2: 2006-2010, 2011-2015, 2016-2020), tumor location (proximal colon, distal colon, rectum), *BRAF* status (wild-type, mutant) and mismatch repair status (proficient, deficient). Patients who died in less than 30 days after surgery were excluded from analysis. p_{trend} values were calculated by using the three ordinal categories of immune cell densities as continuous variables in univariable and multivariable Cox proportional hazard regression models.
